# Preparation and Layer-by-Layer Solution Deposition of Cu(In,Ga)O_2_ Nanoparticles with Conversion to Cu(In,Ga)S_2_ Films

**DOI:** 10.1371/journal.pone.0100203

**Published:** 2014-06-18

**Authors:** Walter J. Dressick, Carissa M. Soto, Jake Fontana, Colin C. Baker, Jason D. Myers, Jesse A. Frantz, Woohong Kim

**Affiliations:** 1 Center for Bio/Molecular Science & Engineering, United States Naval Research Laboratory, Washington, District of Columbia, United States of America; 2 Optical Sciences Division, United States Naval Research Laboratory, Washington, District of Columbia, United States of America; Osaka University, Japan

## Abstract

We present a method of Cu(In,Ga)S_2_ (CIGS) thin film formation via conversion of layer-by-layer (LbL) assembled Cu-In-Ga oxide (CIGO) nanoparticles and polyelectrolytes. CIGO nanoparticles were created via a novel flame-spray pyrolysis method using metal nitrate precursors, subsequently coated with polyallylamine (PAH), and dispersed in aqueous solution. Multilayer films were assembled by alternately dipping quartz, Si, and/or Mo substrates into a solution of either polydopamine (PDA) or polystyrenesulfonate (PSS) and then in the CIGO-PAH dispersion to fabricate films as thick as 1–2 microns. PSS/CIGO-PAH films were found to be inadequate due to weak adhesion to the Si and Mo substrates, excessive particle diffusion during sulfurization, and mechanical softness ill-suited to further processing. PDA/CIGO-PAH films, in contrast, were more mechanically robust and more tolerant of high temperature processing. After LbL deposition, films were oxidized to remove polymer and sulfurized at high temperature under flowing hydrogen sulfide to convert CIGO to CIGS. Complete film conversion from the oxide to the sulfide is confirmed by X-ray diffraction characterization.

## Introduction

Quaternary chalcogenide semiconductors of structure CuA_x_B_1−x_Z_2_ (where A, B = In, Ga or Zn, Sn; Z = S or Se; 0 ≤ x ≤ 1) are among the leading materials candidates under study as absorber layers for conversion of visible and near infrared solar radiation into electricity in photovoltaic devices. These materials offer several important advantages, including composition tunable band gaps for light absorption matched to the solar spectrum, a large knowledge base of their fundamental properties accumulated over decades of research, and photochemical, chemical, and thermal stability, among others. [1]–[5] However, achieving sufficient power/energy conversion efficiencies (*i.e*., >20%) using appropriate materials and systems that can be prepared at low cost remain fundamental barriers to photovoltaic commercialization. [1].

With regard to the latter, a key issue in lowering costs is the ability to prepare high quality materials and films in large quantities using processes amenable for high throughput manufacturing. Although vacuum techniques such as sputtering [6], [7] and co-evaporation [8] offer exquisite control over composition and deposition of semiconductor absorbers, they remain largely more costly processing techniques. Consequently, significant efforts are being expended to develop alternative non-vacuum synthetic and film deposition routes based on liquid phase processes compatible with high throughput manufacturing. These include such diverse well-developed technologies as electrodeposition, [7], [9] sol-gel/chalcogenization, [10] reactive solution deposition, [11] interfacial self-assembly, [12] and spincoating, dipcoating, doctor-blading, or ink printing, [5], [13]–[17] alone or in combination with subsequent thermal annealing treatments.

One increasingly popular liquid phase dipcoating technology compatible with high throughput manufacturing is layer-by-layer (LbL) deposition. [18], [19] LbL films are formed via alternate exposure of a substrate to separate aqueous solutions or dispersions containing oppositely multi-charged species. A surface charge reversal occurs during substrate treatment with each species, allowing controlled conformal electrostatic deposition of the oppositely-charged material during the next step. For example, polymer multilayer films are readily prepared using solutions of cationic and anionic polyelectrolytes, with film thickness, structure, and morphology controlled by pH, [20], [21] added salt type, [22] ionic strength, [20] polyelectrolyte molecular weight, [23] and/or temperature [24] during the deposition process. Replacement of one or both polyelectrolyte solutions by appropriately charged nanoparticle dispersions permits fabrication of composite materials. [18], [25]–[30].

With regards to solar energy applications, the LbL technique has been increasingly exploited for device fabrication, *albeit on smaller scales often constrained by the availability of large amounts of the component nanoparticles*. For example, Lee and coworkers [31] have deposited SnO_2_ nanoparticle/polyallylamine (PAH) multilayers that were sintered to prepare SnO_2_ films useful for cascadal energy band gap matching in dye sensitized solar cells (DSSCs). Furthermore, Ruhlmann and coworkers [32] have recently fabricated DSSCs via a LbL approach using polyoxometalate and porphyrin dye components. Zotti and coworkers [33] have prepared photovoltaic cells from LbL multilayers comprising PbSe nanocrystals and polyvinylpyridine, evaluating semiconductor stability and properties using photoelectrochemical and photoconductivity techniques. In similar fashion, Nozik and coworkers [16] have described LbL deposition of Schottky solar cell devices prepared from PbSe nanocrystals and ethanedithiol crosslinkers, with an unsintered device exhibiting 2.1% efficiency. More recently, Srestha and coworkers [34] have reported a photovoltaic device incorporating a multilayer absorber layer prepared via LbL deposition of polyethylenimine (PEI) and polystyrenesulfonate (PSS)/oleylamine-coated copper-indium-gallium selenide (CIGSe) nanoparticles, demonstrating 3.5% efficiency for the non-optimized device.

We describe here a unique approach using copper-indium-gallium oxide (CIGO) nanoparticles, *which are readily produced in large quantities*, in combination with automatable LbL deposition methods for the low cost manufacture of copper-indium-gallium sulfide (CIGS) films. Our method involves gram scale preparations of CIGO nanoparticles using a flame spray pyrolysis (FSP) technique, [35]–[37] their subsequent surface modification via binding of polyallylamine (PAH), and the formation of stable aqueous dispersions prepared from the resulting CIGO-PAH colloids. Composite multilayer films comprising the CIGO-PAH colloids, together with PSS or polydopamine (PDA), are further prepared via an aqueous LbL approach and characterized. Subsequent oxidation to remove the organic components and sulfurization to form CIGS films for use as potential light absorber layers in photovoltaic devices are also characterized and discussed.

## Experimental Section

### Materials

All chemicals were used as received from the indicated sources unless otherwise noted. Deionized water (18.2 MΩ⋅cm) for all experiments was prepared by passing water through an Elix 5 Milli-Q Plus Ultra-Pure Water System (Millipore Corp.). Nitrogen gas was obtained from in-house liquid N_2_ boil-off and passed through a cellulose filter prior to use. Methane and oxygen gas for flame spray pyrolysis (FSP) experiments and hydrogen sulfide gas for the sulfurization experiments were from Airgas Inc. Acetone, methanol, hydrochloric acid, sulfuric acid, glacial acetic acid, sodium hydroxide (≥ 99.99%, Electronics Grade), sodium chloride, dopamine hydrochloride, tris(hydroxymethyl)aminomethane (Tris), polyethylenimine (PEI, 750,000 g⋅mole^−1^), polyallylamine hydrochloride (PAH, 70,000 g⋅mole^−1^; 15,000 g⋅mole^−1^; 8,500–11,000 g⋅mole^−1^), and polystyrenesulfonate (PSS, 70,000 g⋅mole^−1^) were all ACS Reagent Grade except where otherwise noted from Aldrich Chemical Co. Polyallylamine hydrochloride (PAH, 120,000–200,000 g⋅mole^−1^) was from Alfa-Aesar. Anhydrous ethanol was 200 proof from the Warner-Graham Company. *N*-(2-aminoethyl)-3-aminopropyltrimethoxysilane (EDA, >95%) from Gelest Inc. was purified by vacuum distillation (140–142°C; 14–15 mm Hg). High purity (≥ 99.999%) copper (II) nitrate hydrate, gallium (III) nitrate hydrate, and indium (III) nitrate hydrate from Aldrich Chemical Co. were used for the preparation of copper-indium-gallium oxide (CIGO) in the FSP experiments.

Silicon wafers (100 mm diameter, 500–550 µm thickness, <100> orientation, p-type (B doping), resistivity 6–9 Ω, No. PD7403) from Wacker Siltronic Corp. were cut into 25 mm ×50 mm pieces for use in the experiments. Polished quartz slides (25.4 mm ×50.8 mm ×1 mm) were from Quartz Scientific Inc. Silicon wafers and quartz slides were cleaned per the literature method [38] via successive 30 min immersions in 1∶1 v/v HCl/CH_3_OH and concentrated H_2_SO_4_, with copious water rinsing after each treatment. An EDA self-assembled monolayer (SAM) was chemisorbed onto the freshly cleaned Si wafer and quartz substrate surfaces by treatment for 25 min in 1% (v/v) EDA aqueous solution containing 1 mM acetic acid, followed by a triple water rinse, drying in the filtered N_2_ gas stream, and a 6 min bake at 110°C to complete the chemisorption process. [39].

Molybdenum foil (≥ 99.9%, Aldrich Chemical Co., 50 mm ×50 mm ×1 mm) substrates were cut into 25 mm ×50 mm pieces for use in experiments and then degreased by successive 5 min rinses in acetone, methanol, and water. The substrates were next polished to remove surface oxides and stains using 15 µm alumina grit followed by 3 µm diamond grit. The polished Mo substrates were rinsed successively with water, isopropanol, and acetone, followed by sonication in water for 15 min at 80 W power using a Branson Model 2510 Ultrasonic Cleaner/Water Bath to dislodge any polishing grit adhering to the surface before drying in a filtered N_2_ gas stream. The Mo substrates exhibited a near mirror finish with very faint visible scratches after processing. EDA-coated Si wafers and quartz substrates and polished Mo substrates were each stored in Fluoroware containers until need for experiments. The stored, polished Mo substrates were soaked 1 h in 1/1 v/v HCl/methanol and rinsed with water immediately before deposition of multilayer films. The EDA-coated quartz slides and Si wafers were removed from storage and used directly for multilayer depositions.

### Instrumentation

Sonication reactions of polyelectrolyte and CIGO particles were carried out using a Sonicators & Materials Inc. Vibra-Cell Sonicator equipped with a titanium horn. All pH measurements were made using a Corning Pinnacle Model 530 pH meter. A Sorvall Model RC5B Plus Refrigerated Ultracentrifuge equipped with a Sorvall Model SS-34 rotor was used for all centrifugations. CIGO-PAH pellets formed during centrifugation were re-dispersed in water using a Scientific Industries Inc. Model Vortex-2 Genie vortexer. Freeze drying experiments were performed using a VirTis Inc. benchtop K freeze dryer. Automated deposition of some films was carried out using a StratoSequence VI Robot Dipcoater from nanoStrata Inc.

UV-visible absorbance spectra were acquired using a double beam Varian Cary 5000 spectrophotometer. Film spectra were referenced to an EDA-coated quartz slide baseline. Absorbance spectra of various solutions and the CIGO-PAH dispersion were acquired using the same instrument with 0.10 cm pathlength quartz cells referenced to a water blank baseline. Thermogravimetric measurements of the CIGO and CIGO-PAH particles were made using a TA Instruments Inc. Hi-res TGA2950 Thermogravimetric Analyzer. CIGO-PAH dispersions were characterized using a NanoSight LM10-HSBF nanoparticle characterization system along with corresponding software Nanosight NTA 2.2 (www.nanosight.com; Nanosight Worthington, OH, USA) for particle concentration determinations and a Brookhaven Instruments ZetaPALS dynamic light scattering (DLS) system equipped with 1 cm path-length cuvettes for particle size measurements.

X-Ray Photoelectron Spectroscopy (XPS) measurements was acquired using a Thermo Scientific K-alpha XPS system equipped with Al k-α source at 1486 eV. A sample spot size of 400 µm was used. Scanning electron microscopy (SEM) was performed using a Carl Zeiss SMT Supra 55 electron microscope with a Princeton Gamma Tech EDS detector for compositional analysis of films. A Scintag XDS 2000 diffractometer using Cu-kα radiation and Rigaku SmartLab x-ray diffractometer using Cu-Kα radiation were used to collect x-ray diffraction spectra for phase identification of the as-prepared CIGO particles and CIGS films, respectively. A KLA-Tencor AlphaStep D-120 profilometer was used to measure the thickness of the various as–prepared, oxidized, and sulfurized films. A custom Kurt J. Lesker Octos cluster tool was used for film sputtering and electron beam evaporation in the preparation of the photovoltaic test device. A JEOL JEM-2200FS field emission electron microscope was used to image the as-prepared CIGO particles dropcast as an ethanol suspension onto a SPI 200 mesh holey carbon coated Cu TEM grid.

### CIGO Particle Preparation

CIGO nanoparticles of composition CuIn_x_Ga_1−x_O_2_ (0 ≤ x ≤ 1) were prepared using ethanol stock solutions containing appropriate ratios of the copper, indium, and gallium nitrate precursors. For the x ≅ 0.7 composition (best matched to the solar spectrum upon conversion to CIGS) of interest here, a stock solution was prepared by dissolving 20.0 g (0.1066 moles, anhydrous) copper (II) nitrate hydrate, 23.28 g (0.0774 moles, anhydrous) indium (III) nitrate hydrate, and 9.16 g (0.0358 moles, anhydrous) gallium (III) nitrate hydrate in 200 mL ethanol. The stock solution was fed through a homemade flame spray apparatus nozzle at flow rates of 5 mL⋅min^−1^ with the aid of an O_2_ dispersion gas/oxidant under a flow rate of 5 L⋅min^−1^. Small pilot flames ignited from flowing 1.5 L⋅min^−1^ CH_4_ and 3 L⋅min^−1^ O_2_ and forming a ring pattern were used as the flame ignition source and as a supporting flame for the larger central flame. The pilot flame ringlet surrounded a central capillary tube that sprayed the precursor solution mixed with oxygen dispersant gas to form precursor droplets that underwent combustion in the large central flame. The CIGO powders were either deposited directly on Mo-coated sodalime glass substrates heated by the flame to 400°C or collected on glass fiber filter paper mounted in a water cooled stainless steel collection chimney. For the case of deposition on the filter media the powders were removed by scraping with a Teflon spatula.

### Polyelectrolyte Binding to CIGO

The following general procedure, illustrated for PAH (molecular weight 8,500–11,000 g⋅mole^−1^), was used for reaction of all amine polyelectrolytes with the CIGO particles. The procedure for reaction of PSS was identical, with the exception that the PSS solution pH was 6.8 rather than the pH 8.2−8.3 used for polyamines.

A 5 mg PAH⋅mL^−1^ stock solution was prepared by dissolving 1.250 g PAH in 230 mL 1.00 M NaCl (aq) solution in a beaker with stirring. A freshly prepared 6 M NaOH (aq) solution was added dropwise with stirring until the pH = 8.3±0.1. The solution was transferred to a 250 mL volumetric flask, diluted to the mark with 1.00 M NaCl (aq) solution, and stored in a sealed glass bottle under N_2_ atmosphere until needed for experiments. The 5 mg PAH ⋅mL^−1^ 1.00 M NaCl (aq) solution had a pH = 8.2±0.1. Similar stock polyelectrolyte solutions were prepared using PEI and PSS (at pH 6.8) in 1.00 M NaCl (aq) as necessary.

A 125 mg sample of CIGO powder and 80 mL of stock 5 mg PAH⋅mL^−1^ 1.00 M NaCl (pH 8.2) solution were added to a 150 mL high walled Pyrex beaker (80 mm height ×55 mm diameter) resting on a lab jack and securely clamped in place in a well-ventilated fume hood. The height of liquid in the beaker was 35 mm. The sonicator horn was immersed into the mixture such that the bottom of the horn was fixed 18−20 mm above the bottom of the beaker, raising the liquid height to 40 mm. The horn assembly was securely clamped in place and the beaker was loosely wrapped with an Al foil cone extending 10 cm above the top of the beaker as a splash guard. The sonicator power was set to 480 W and the mixture was sonicated for 30 min (***Caution*** **:** noise hazard- ear protection required). Following sonication, the horn and Al foil cone were quickly removed and the temperature of the gray-black dispersion was measured by thermometer. A temperature of 50−55°C was routinely measured, consistent with heating during sonication that reduced the dispersion volume to 60–65 mL.

The dispersion was allowed to cool to room temperature for 20 min, transferred (equal weights) into two centrifuge tubes, and centrifuged for 20 min at 10,000 rpm and 4°C. The clear, blue-green supernatant containing unreacted excess PAH/1.00 M NaCl (aq) solution was decanted and discarded, leaving a gray-black pellet of PAH-modified CIGO particles. Wash water was added to each centrifuge tube in amounts required to provide equal tube weights, the tubes were resealed, and the CIGO-PAH pellet was re-dispersed using a vortexer. The samples were re-centrifuged for 20 min at 10,000 rpm (4°C) and the clear, colorless wash water was discarded from the pellet to complete the first wash cycle. The CIGO-PAH pellet was subjected to 3 such additional wash cycles.

After the final wash, weight measurements indicated that 45–50 mg CIGO-PAH were lost during processing, leaving 75–80 mg CIGO-PAH in the final pellets. The pellets were combined, re-suspended in 80 mL water to provide a 1 mg CIGO-PAH⋅mL^−1^ aqueous stock dispersion, and stored in tightly sealed BLUE MAX 50 mL Falcon polypropylene conical tubes until needed for film depositions. Unused 1 mg CIGO-PAH⋅mL^−1^ aqueous stock dispersions were typically discarded after 6 days and replaced by freshly prepared dispersions for film depositions unless noted otherwise.

### CIGO-PAH Particle Concentration Determination

Freshly prepared 1 mg CIGO-PAH⋅mL^−1^ aqueous dispersion was diluted in a series with water until concentrations in the optimal 2–4×10^8^ particles⋅mL^−1^ range observable by the NanoSight LM10-HSBF nanoparticle characterization system were obtained. A 60 sec video of particle Brownian motion was taken at room temperature and analyzed to obtain a particle count using the Nanosight NTA 2.2 software. Brightness and gain were the same for all samples and the detection threshold was automatically adjusted by the software. A blur size of 5 pixel ×5 pixel was chosen for all samples. CIGO-PAH particle concentration in the original sample was then calculated from the dilution factors (1/2000) used and the recorded particle counts.

### CIGO-PAH Particle Size and Stability Determination

A 3 mL aliquot of 1 mg CIGO-PAH⋅mL^−1^ aqueous dispersion contained in a 1.00 cm pathlength cuvette was used for each dispersion particle size and stability determination. Ten DLS measurements were taken and averaged in any time point at 22°C. Two stability studies were performed. For long term stability determination, data was acquired once a day until day 13 for the dispersion. A second stability study was conducted for 1 mg CIGO-PAH⋅mL^−1^ aqueous dispersion containing 20 mM Tris (pH 8.25) buffer. For this study, 60 µL of 1.00 M Tris pH 8.25 aqueous buffer was mixed with 3 mL of the freshly prepared 1 mg CIGO-PAH⋅mL^−1^ aqueous dispersion. Data was collected continuously for a period of 2 h, after which measurements were taken every 30 min for a period of 8 hr. All samples were kept at room temperature and ambient pressure during the experiments.

### Fabrication of PSS/CIGO-PAH Multilayer Films

Sealable, grooved plastic containers designed for storage of microscope slides were used as sample containers for the quiescent hand dipcoated LbL PSS/CIGO-PAH multilayer depositions. Containers were securely positioned such that substrates were vertically oriented during depositions to minimize the chances of binding aggregates or precipitates formed during treatments. Sufficient quantities of 1 mg CIGO-PAH⋅mL^−1^ dispersion and 5 mg PSS⋅mL^−1^ 1.00 M NaCl (aq) solution to cover the substrates (*i.e*., EDA-coated Si wafers and quartz slides) to a depth of 25–30 mm were placed in separate containers. EDA-coated Si wafers (*i.e*., Si-EDA) and EDA-coated quartz slides (*i.e*., Q-EDA) were then placed directly into the PSS solution and the container was sealed. After 30 min, the substrates were removed and rinsed for 60 s each with agitation in water. The substrate rinse was repeated two additional times in fresh water before drying in the filtered N_2_ gas stream.

Samples were then transferred to container holding the CIGO-PAH dispersion for deposition of the CIGO-PAH nanoparticles. Samples were typically treated for 5−6 h at room temperature. Overnight treatments (*i.e*., ≥ 12 h) resulted in no further appreciable particle deposition (*i.e*., <2–3%), as measured by UV absorbance spectroscopy at 225 nm or 285 nm. Following the CIGO-PAH treatment, the samples were washed and dried as described for the PSS treatment. This cycle was repeated to build PSS/CIGO-PAH multilayers on the substrate surface.

For the Mo substrate, a polydopamine (PDA) coating was deposited prior to deposition of CIGO-PAH and PSS. Polymerization of dopamine was initiated by addition of a 100 µL aliquot of 1.00 M Tris pH 8.25 (aq) buffer to 10 mL of a freshly prepared 1 mg dopamine hydrochloride⋅mL^−1^ (aq) solution. The Mo substrate was treated with this solution for 70 min, then removed, rinsed thoroughly three times in fresh water, and dried in the filtered N_2_ gas stream. The PDA-coated Mo substrate was then treated as described below with a 1 mg CIGO-PAH⋅mL^−1^ 20 mM Tris pH 8.25 dispersion to initiate the multilayer deposition. Alternating treatments with the 5 mg PSS⋅mL^−1^ 1.00 M NaCl (aq) solution and 1 mg CIGO-PAH⋅mL^−1^ dispersion were then performed as described above until the multilayer film of desired thickness had been deposited.

For multilayers fabricated using the robot dipcoater, 45–50 mL aliquots of 1 mg CIGO-PAH⋅mL^−1^ dispersion and 5 mg PSS⋅mL^−1^ 1.00 M NaCl solution were placed into separate treatment beakers. The robot controls were set to refill each of 6 rinse beakers with 70 mL water after each use. Substrates were loaded onto the sample holder and spun during treatments and rinses at 140±5 rpm. Substrates were treated with PSS solution for 30 min and CIGO-PAH dispersion for 90 min, which was the maximum treatment time allowable. The substrates were rinsed 60 s in each of 3 beakers containing fresh water and dried for 90 s in a stream of filtered N_2_ gas following deposition of each PSS or CIGO-PAH layer. The CIGO-PAH dispersion and PSS solution were replaced and the sample and rinse beakers thoroughly rinsed with water and dried after deposition of every 4−6 PSS/CIGO-PAH bilayers. Multilayers fabricated using the robot dipcoater were left standing in air in the closed sample chamber following completion of a deposition cycle until the initiation of the next cycle.

For hand dipcoated multilayers comprising large numbers of PSS/CIGO-PAH bilayers requiring fabrication times of days or weeks, samples bearing partially complete films were stored in PSS solution overnight (*i.e*., 12−16 h). Because PSS depositions were essentially complete (*i.e*., 100%) within 30 min under our conditions, no further PSS deposition occurred overnight. The absorbance spectra of PSS/CIGO-PAH multilayers on Q-EDA substrates were periodically recorded as the depositions proceeded to monitor film growth. CIGO-PAH dispersion and PSS solution were replenished with fresh aliquots after deposition of every 4−6 PSS/CIGO-PAH bilayers. Completed PSS/CIGO-PAH films were stored in sealed Fluoroware containers until needed for additional experiments. Sample containers holding the CIGO-PAH dispersion and PSS solutions were cleaned after completion of film depositions by soaking overnight in 6 M HCl (aq) solution, which completely dissolved the CIGO-PAH particles, and water, respectively, followed by copious rinsing with water and drying in the filtered N_2_ gas stream prior to re-use.

### Fabrication of PDA/CIGO-PAH Multilayer Films

The preparation of the PDA/CIGO-PAH multilayers proceeded in similar fashion to the fabrication of the PSS/CIGO-PAH films with the changes noted below. First, freshly prepared PDA was formed *in situ* during substrate treatment by the polymerization of dopamine in basic aqueous solution for the deposition of each PDA layer. A stock aqueous solution containing 1 mg dopamine HCl⋅mL^−1^ was prepared and stored sealed in the dark at 2−4°C for up to 3 days until needed. A 10 mL aliquot of the stock dopamine HCl solution was allowed to warm to room temperature during 5–10 min, after which 100 µL of 1.00 M Tris pH 8.25 (aq) buffer was added with mixing to initiate PDA polymerization. The substrates to be treated were immediately placed in the solution and allowed to stand for 45 min, during which time the solution developed a yellow-brown color. The substrates were removed from the PDA solution, which was discarded, and rinsed and dried as described for the PSS/CIGO-PAH multilayers. The container holding the PDA solution was rinsed thoroughly with water and dried before re-use for the next PDA layer deposition using freshly prepared PDA. The container was cleaned after completion of film depositions by immersion in KOH-saturated isopropanol for 30 min, followed by copious rinsing with water and drying in the filtered N_2_ gas stream prior to re-use.

Second, the 1 mg CIGO-PAH⋅mL^−1^ aqueous dispersion was made basic by addition of 150 µL 1.00 M Tris pH 8.25 buffer to 7.5 ml of the CIGO-PAH dispersion immediately prior to use for film depositions. The PDA-coated substrates were treated 45 min with this CIGO-PAH/Tris pH 8.25 dispersion. Each CIGO-PAH/20 mM Tris pH 8.25 dispersion aliquot was used for fabrication of 2–3 PDA/CIGO-PAH bilayers before replacement with a fresh dispersion aliquot. Lastly, unlike the PSS/CIGO-PAH multilayers, PDA/CIGO-PAH multilayers terminated with a CIGO-PAH layer were stored dry overnight in Fluoroware containers until depositions resumed the next day.

### Film Oxidation, Sulfurization, and Photovoltaic Test Device Fabrication

PSS/CIGO-PAH and PDA/CIGO-PAH multilayer films on Si-EDA, Q-EDA, and Mo substrates were thermally annealed 5 h at 550°C in air to remove organic components such PSS, PAH, and PDA and complete the oxidation of the CIGO particles in the films. The oxidized films were converted to the corresponding CIGS films via sulfurization by thermal treatment at 550°C in a flowing 20 ccm H_2_S gas stream for 3 h. After film oxidation and sulfurization, the resulting CIGS absorber films were characterized as described in the text.

A CIGS film, prepared by oxidation and sulfurization of a 46 bilayer PDA/CIGO-PAH film deposited on a Mo substrate, was used to prepare photovoltaic devices. This CIGS film was coated with a CdS buffer layer via chemical bath deposition per the literature method. [40] A ZnO/aluminum-doped ZnO (2%) film was then applied to the CdS layer via RF magnetron sputtering, followed by electron beam evaporation of Ni/Al grids to complete the photovoltaic device. [41] Device areas were mechanically scribed to 0.5 cm^−2^. Devices were illuminated under a calibrated 100 mW⋅cm^−2^ AM1.5G solar simulator for current-voltage characterization.

## Results and Discussion

### CIGO Particles

FSP is a non-vacuum gas phase process capable of producing high purity oxide nanoparticles on an industrial scale. [35]–[37] Volatile or aerosolized liquid fuel containing nanoparticle precursor materials is sprayed into a flame, where the droplets evaporate and undergo combustion. The species formed are rapidly quenched as they leave the reaction zone and deposit as nanoparticle oxides on a cold collector surface. We have for the first time successfully produced gram scale quantities of copper-indium-gallium oxide (CIGO) nanoparticles, having an average composition CuIn_x_Ga_1−x_O_2_, using the technique from ethanol solution containing metal nitrate precursors. Although nanoparticles having compositions with 0 ≤ x ≤ 1 can be prepared, we limit our attention here to nanoparticles having x ≅ 0.7 (as the absorbance of CIGS films of similar composition are well matched to the solar spectrum).

**Figure 1:**
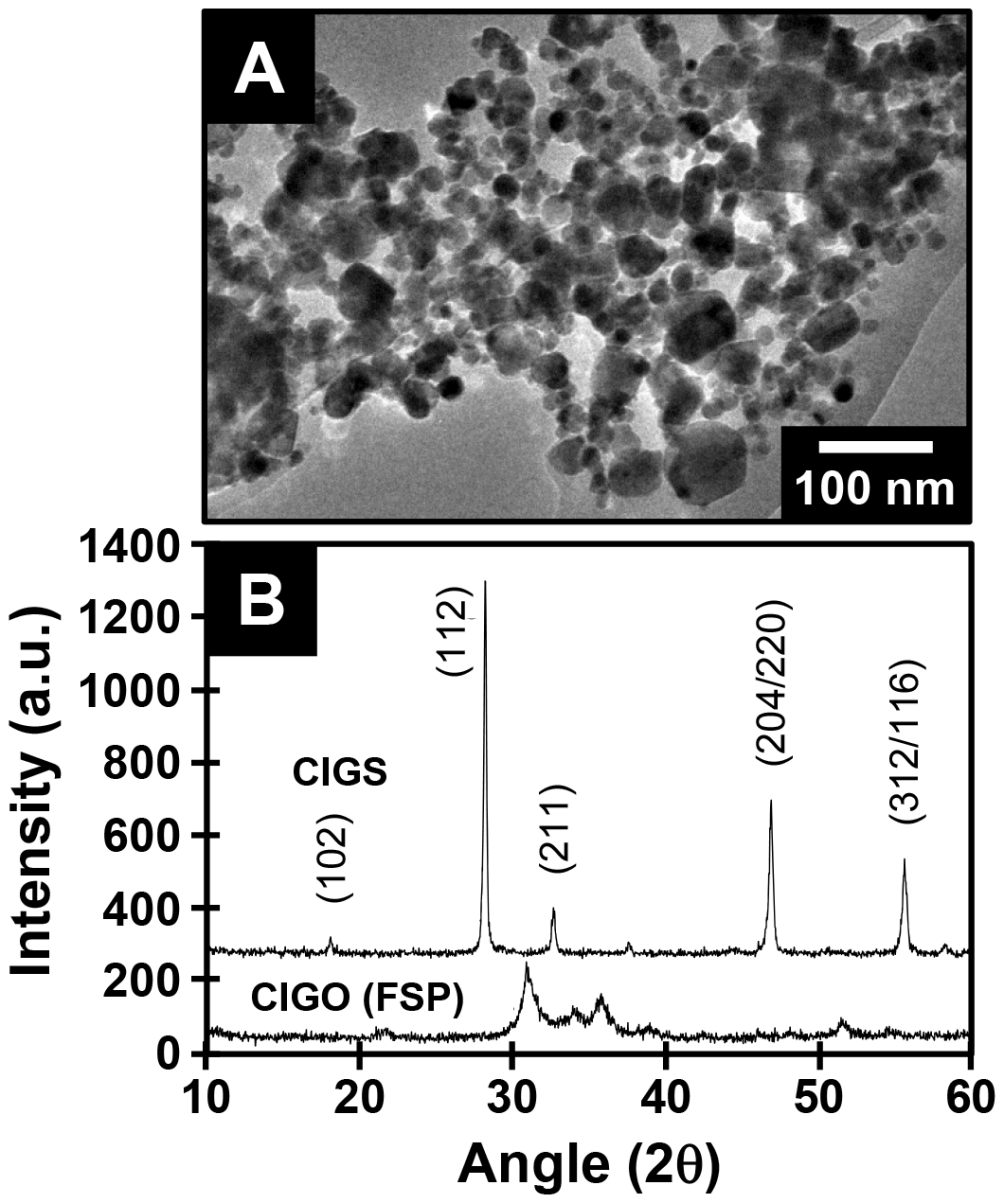
CIGO nanoparticle characterization. (A) TEM of FSP as-prepared CIGO nanoparticles. (B) XRD patterns for the CIGO sample prepared by FSP in part (A) and CIGS species obtained after its sulfurization.

Figure 1, part A, shows the TEM of the as-prepared CIGO nanoparticles. Loose aggregates comprising 10−75 nm diameter particles are typically observed. XRD results in Figure 1, part B, reveal broadened peaks and peak positions consistent with a mixture of oxides, rather than a single phase for the as-formed CIGO particles. Thermogravimetry of the as-formed CIGO particles results in a *weight gain* of 2% during heating to 600°C (Figure S1, part A), consistent with the presence of mixed oxides and/or incompletely oxidized metal species within the particles. Nevertheless, sulfurization readily transforms this material into the corresponding CIGS species, as confirmed by its characteristic XRD pattern [42] in Figure 1, part B. We estimate a density of ρ ≅ 4.0±0.4 g⋅cm^−3^ for our as-prepared CIGO particles from a volume displacement measurement using a fixed mass of material. Consequently, the as-formed particles do not form stable aqueous suspensions as required for use in layer-by-layer film fabrication processes. Therefore, as a first step in the use of these particles for film fabrication we adapted a literature method [43] for stabilizing our aqueous CIGO particle dispersions.

### CIGO-PAH Particle Preparation

Amine ligands such as oleylamine are often used as components during thermal syntheses of semiconductor and metal nanoparticles. [44]–[49] Amines readily bind to metal ion surface sites on the growing nanoparticle, controlling nanoparticle size and providing nanoparticle stability during subsequent particle dispersion in solvents. [45], [47], [48] However, because our nanoparticles were prepared via FSP, incorporation of amine ligand during nanoparticle formation is not possible. Therefore, we instead have investigated thermally activated amine ligand binding to our as-formed particles.

We select polyamines, rather than monomeric amines like oleylamine, for further study for three reasons. First, polyamines provide the potential for more rapid surface binding by virtue of their larger numbers of amine sites, with unbound amine sites remaining available for interaction with water to stabilize the particle dispersion. Second, the ability to control surface charge density of the bound polyamine via solution pH changes provides the requisite cationic surface charge necessary for particle use in the electrostatic LbL film assembly process, while also further enhancing particle dispersion stability. Finally, the ability to control thickness of the bound polyamine via changes in polymer molecular weight and solution ionic strength provides a deformable coating expected to facilitate particle packing during film deposition. [25], [26].

Initial work focused on polyethylenimine (PEI), a high molecular weight (750,000 g⋅mole^−1^) branched polyamine, as the CIGO particle coating. Lvov and coworkers [43] have shown that sonication of colloids in the presence of polyelectrolytes provides the necessary thermal activation for efficient attachment of polyelectrolyte to the colloid particle surface. In addition, sonication agitates and suspends the particles during treatment to promote deposition of a more uniform polyelectrolyte coating.

A sonication treatment was carried out using PEI in 1.00 M NaCl (pH 8.2) solution to promote deposition of thicker films onto the particle surface. Thicker films are deposited from solutions of higher ionic strength because increased counterion pairing with protonated amine sites at higher salt concentrations partially neutralizes polyelectrolyte charge. [22] As repulsive electrostatic interactions among polymer protonated amine sites decrease, van der Waals and related forces become more important leading to polymer chain coiling. Binding of such coiled chains to the particle surface creates a thicker polymer coating on the nanoparticle due to steric repulsions between chains of adjacent polymers, providing additional unbound amine sites for interaction with solvent as required to stabilize particle dispersions. In contrast, extended chains occurring at low ionic strength due to electrostatic repulsions among adjacent protonated amine sites in the polymer produce thinner films with fewer unbound amine sites capable of providing stabilizing interaction with the solvent.

Various experiments under different sonication conditions indicated that 30 min sonication treatment of CIGO particles at 480 W power in aqueous PEI/1.00 M NaCl (pH 8.2) solution was sufficient to bind PEI to the CIGO surface, as evidenced by formation of a stable CIGO-PEI dispersion. Although the temperature of the particle dispersion after sonication was typically 50–55°C, sonication can produce local temperatures ranging from 100s-1000s of degrees which are more than sufficient to promote the amine binding reaction. Cooled dispersions exhibited decreases in liquid volume of 20–25% consistent with heating and appeared gray in color.

During subsequent centrifugation to remove and rinse excess PEI/1.00 M NaCl (pH 8.2) solution from the CIGO-PEI particles, we noted a high degree of particle aggregation indicated by difficulty in re-dispersing the pellet in the wash water. This tendency was further exacerbated if the CIGO-PEI particles were freeze dried prior to re-dispersion. We attributed this behavior to (1) potential non-covalent interactions, such as amine-amine hydrogen bonding and chain entanglements, between extended PEI polymer chains on adjacent particles, and (2) covalent bridging of adjacent nanoparticles by a single surface bound PEI chain. We therefore hypothesized that replacement of branched PEI by a linear polyamine, such as polyallylamine (PAH), of lower molecular weight would minimize aggregation. In fact, re-dispersion of CIGO-PAH particles in water was facilitated relative to CIGO-PEI particles, with particles modified by lower molecular weight PAH generally more readily dispersed consistent with this hypothesis. After a series of experiments, we found that in our hands PAH of molecular weight 8,500−11,000 g⋅mole^−1^ yielded readily dispersible particles during processing that also remained sufficiently stable as aqueous dispersions for subsequent work (*vide infra*). Amine binding to the CIGO particle surface was confirmed by a control experiment in which PSS replaced PAH during sonication. CIGO particles treated with PSS rapidly settled following sonication, consistent with the negligible metal ligating ability of the PSS sulfonate group compared to the PAH amine group.

### CIGO-PAH Particle Dispersion Characterization

We have characterized the CIGO-PAH particles by several techniques to assess their stability and suitability for LbL deposition. The presence of a PAH coating on the CIGO particles is inferred by their formation of a stable aqueous dispersion and electrostatic binding of the particles to a PSS film deposited onto an EDA-coated quartz slide (*vide infra*). In contrast to the 2% weight gain observed for bare CIGO particles, thermogravimetry indicates a 6% weight *loss* for CIGO-PAH particles consistent with oxidation and loss of the organic coating during a temperature ramp to 550°C (Figure S1, part B).

**Figure 2:**
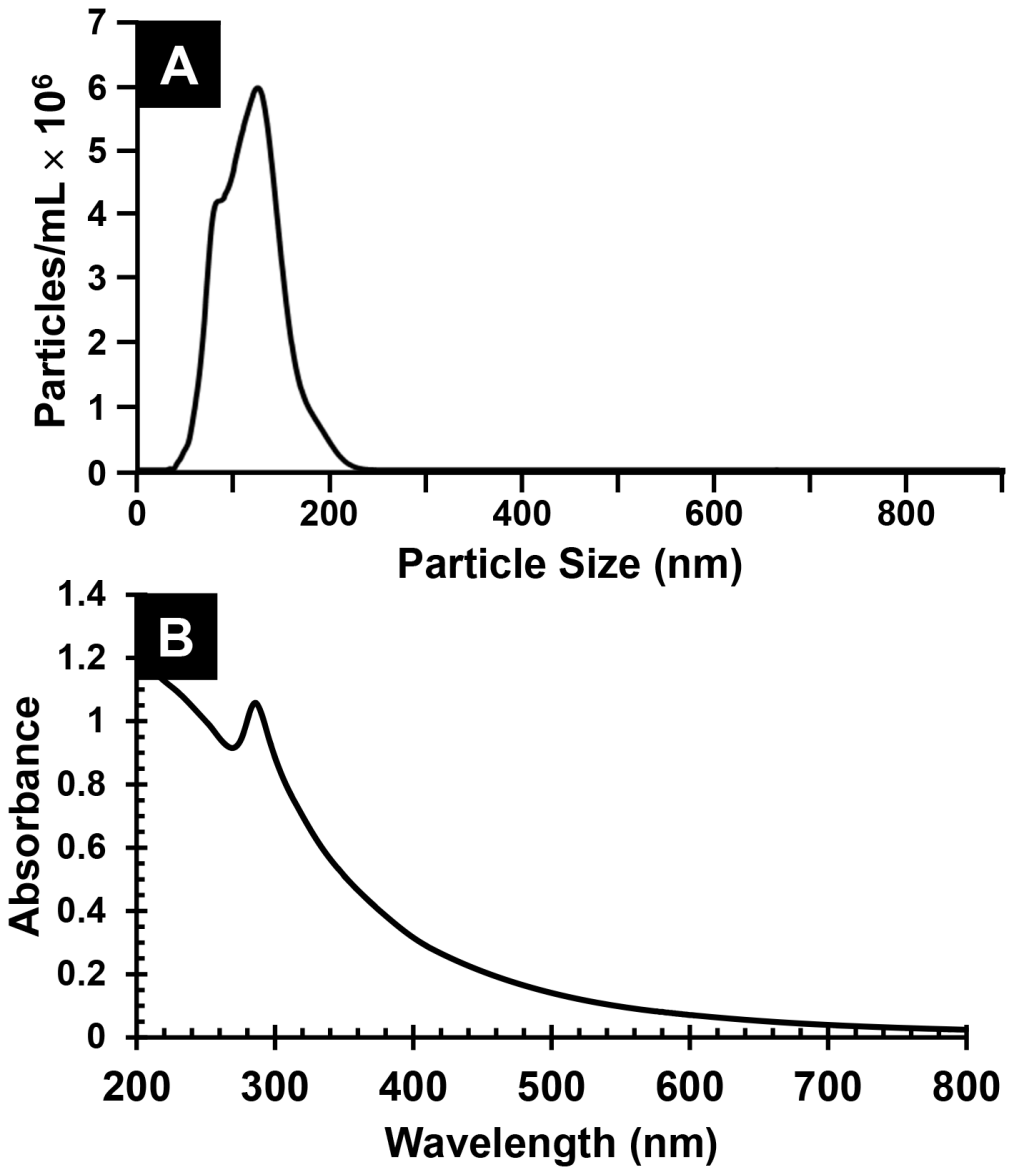
CIGO-PAH dispersion characterization. (A) Particle size distribution and concentration for the fresh 1 mg CIGO-PAH⋅mL^−1^ (aq) dispersion determined using the NanoSight LM10-HSBF nanoparticle tracking system. (B) UV-visible absorbance spectrum of a freshly prepared 0.33 mg CIGO-PAH⋅mL^−1^ aqueous dispersion in a b = 0.10 cm pathlength cuvette *vs*. a water baseline. The peak at 285 nm corresponds to incompletely oxidized species within the particles.

Measurements of Brownian motion of CIGO-PAH particles dispersed in water, made using a NanoSight LM10-HSBF nanoparticle tracking system, indicate that a 1 mg CIGO-PAH⋅mL^−1^ dispersion contains (9±2) ×10^11^ particles⋅mL^−1^ (n = 8), with an average particle size of 125±15 nm and particle distribution shown in Figure 2, part A. While light scattering results measure the total particle diameter comprising the CIGO particle and PAH coating, the 125±15 nm particle size noted for the CIGO-PAH particles compared to the as-prepared 10–75 nm diameter CIGO particles (Figure 1, part A) suggests that some PAH-assisted particle aggregation occurs during sonication despite the use of the lower molecular weight PAH species. The UV-visible absorbance spectrum corresponding to the CIGO-PAH dispersion, shown in Figure 2, part B, is characterized by the rapidly rising absorbance with decreasing wavelengths expected for particle light scattering behavior. The spectrum also features a distinct peak at 285 nm, which is not present in the thermally annealed CIGO particles. Because PAH exhibits no absorbance at wavelengths ≥ 200 nm, we assign this peak to incompletely oxidized species consistent with the weight *gain* observations during thermogravimetric oxidation of the as-prepared CIGO samples (*vide supra*).

**Figure 3:**
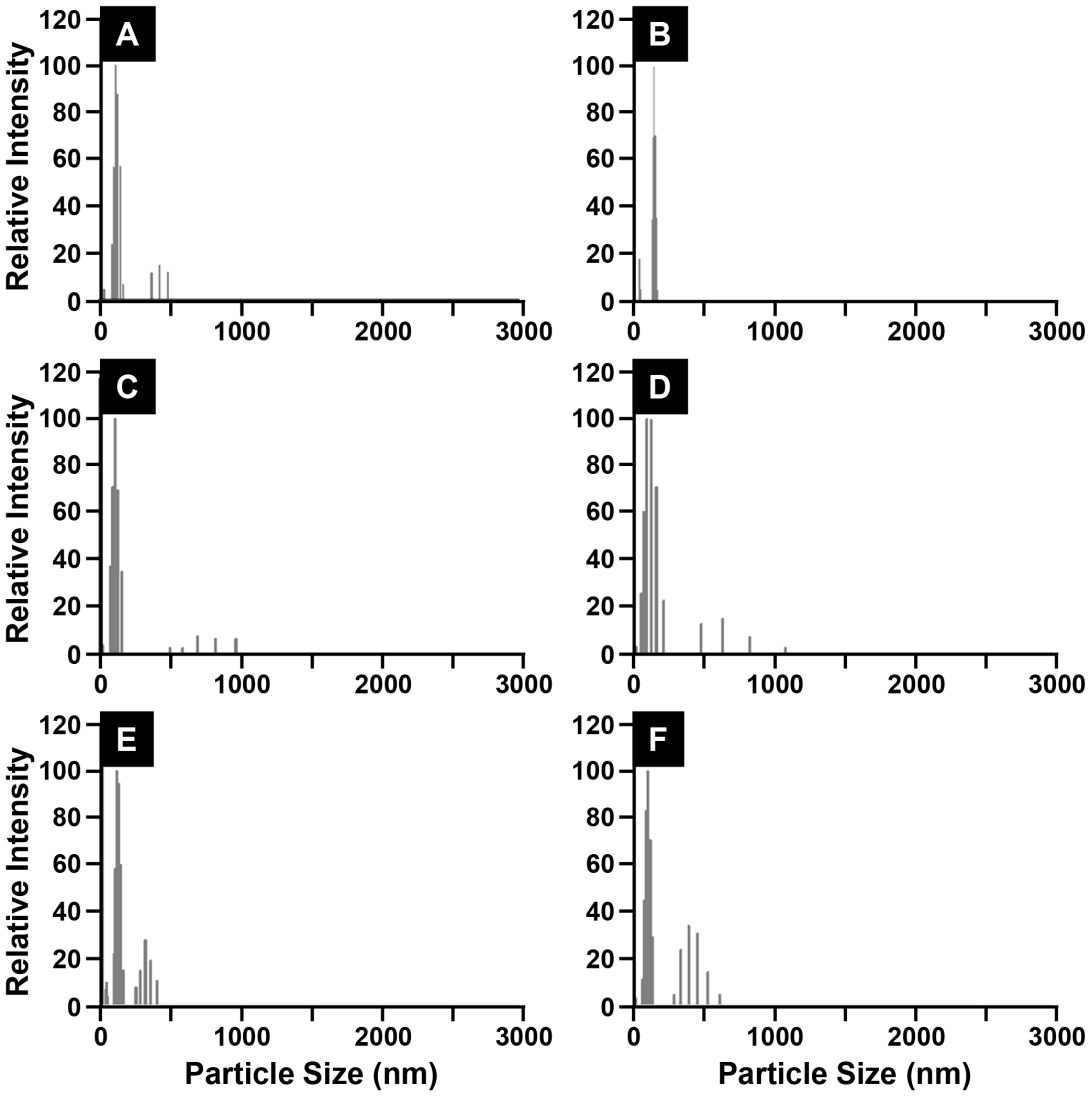
Aqueous CIGO dispersion stability. Particle size dispersion histograms from DLS illustrating the time dependent aggregation of the quiescent 1-PAH⋅mL^−1^ (aq) dispersion are shown. (A) aged 3 days; (B) aged 3 days, after mixing; (C) aged 6 days, 10 min after mixing; (D) aged 6 days, 40 min after mixing; (E) aged 7 days, 10 min after mixing; (F) aged 13 days, 10 min after mixing. Particles larger than 300 nm were set as the aggregation threshold.

Stability of the CIGO-PAH dispersion was also assessed as a pre-requisite for its use in the fabrication of PSS/CIGO-PAH and PDA/CIGO-PAH multilayer films. For the CIGO-PAH dispersions used in the preparation of PSS/CIGO-PAH films, changes in particle size distribution over a 13 day period were monitored by dynamic light scattering (DLS). The appearance of particles larger than 300 nm, which were not observed in the freshly prepared dispersion, was set as the aggregation threshold. Aggregates were detected in the dispersion prior to mixing only after 3 days, as shown in Figure 3, part A. However, aggregates were no longer detected upon mixing the sample, indicating that aggregation at this stage was still reversible (Figure 3, part B). In contrast, after 6 days no aggregates were detected in the quiescent sample (Figure S2) until the sample was mixed. Aggregates were then detected 10 min after mixing, as shown in Figure 3, part C, and persisted for at least 40 min (Figure 3, part D) indicative of irreversible aggregation. Even larger aggregates were detected at days 7 and 13 after mixing followed by a 10 min wait, consistent with further destabilization of the dispersion as shown in Figure 3, parts E and F. DLS measurements indicated that <5% of the original particles separated from the dispersion as a result of settling during the course of the experiment, a value that did not materially alter particle binding kinetics under our deposition conditions.

**Figure 4:**
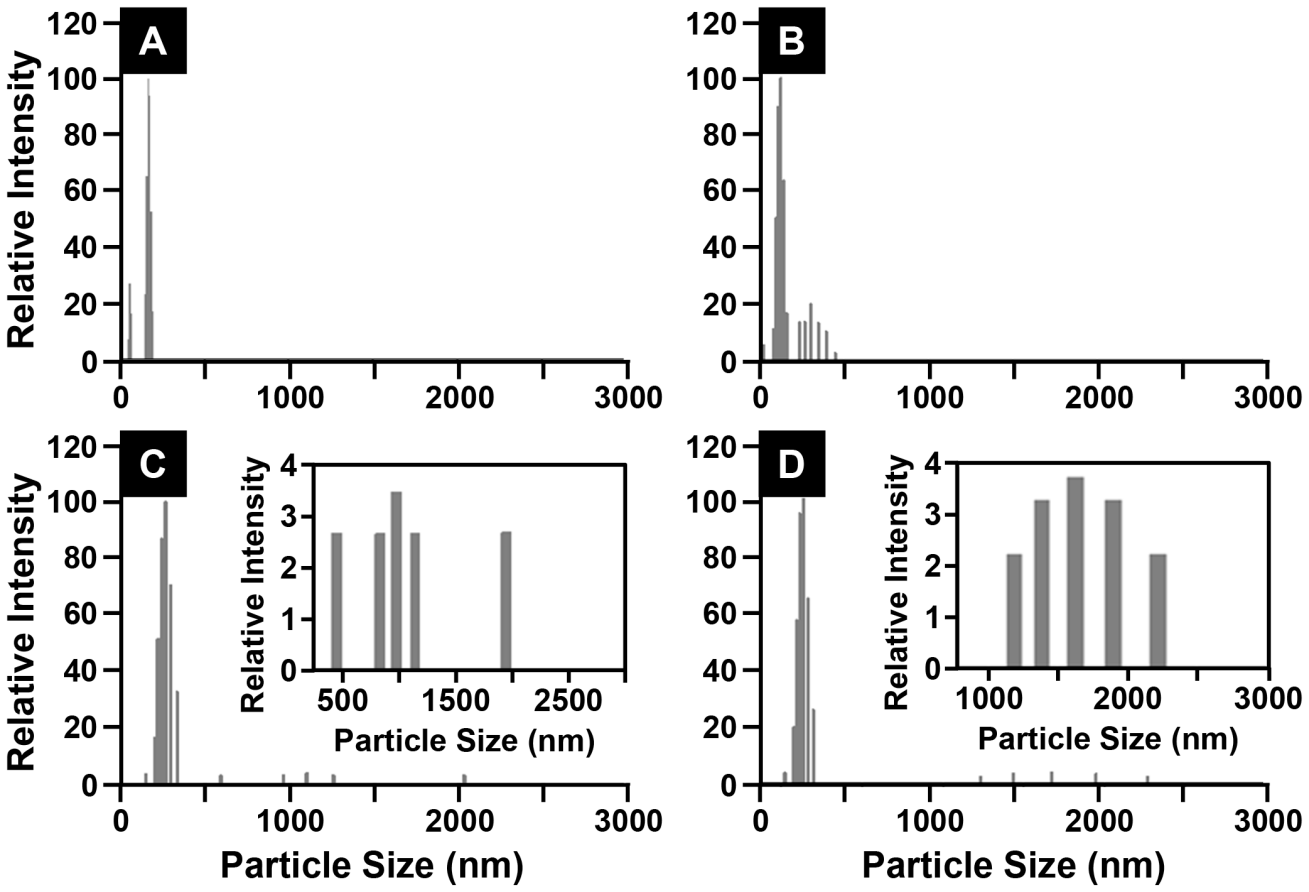
Aqueous CIGO dispersion stability in pH 8.25 buffer. Particle size histograms from DLS illustrating the time dependent aggregation of the 1-PAH⋅mL^−1^ 20 mM Tris pH 8.25 (aq) dispersion are shown. (A) At preparation (t = 0 min). (B) t = 30 min after preparation. (C) t = 50 min after preparation. (D) t = 420 min after preparation. Insets in parts (C) and (D) are expansions of the particle distributions for particles larger than 400 nm diameter.

The stability of freshly prepared aqueous 1 mg CIGO-PAH⋅mL^−1^ 20 mM Tris pH 8.25 buffered dispersion used for the deposition of the PDA/CIGO-PAH films was also evaluated, under quiescent conditions, by DLS measurements during an 8 h aging experiment. The average size of the most abundant particles in the dispersion during 8 h was 137±22 nm (n = 22). Some particles larger than 300 nm were first detected by DLS after 30 min and after 50 min some micron size particles were observed, as shown in Figure 4. Aggregation occurred in periodic fashion in which micron size particle levels initially increased to a threshold value, then dropped precipitously as the large aggregates settled out of the dispersion before the cycle began anew. Quantities of micron sized aggregates were detected after 50, 110, 220, and 420 min of aging, with few if any noted at intermediate times consistent with this mechanism. The growth and presence of micron size particles confirmed that Tris pH 8.25 buffer promotes particle aggregation as the dispersion ages, behavior consistent with partial PAH deprotonation (pK_a_ ≅ 9.5–10) at pH 8.25 leading to loss of stabilizing positive charge sites on the CIGO-PAH particle. In contrast to the CIGO-PAH dispersion study, a somewhat larger fraction of particles were removed from the CIGO-PAH/Tris pH 8.25 dispersion (<10%; Table S1) via aggregation and settling. Nevertheless, the CIGO-PAH/Tris pH 8.25 dispersion remained sufficiently stable for use in film fabrication under our deposition conditions.

### PSS/CIGO-PAH and PDA/CIGO-PAH Multilayer Depositions

**Figure 5:**
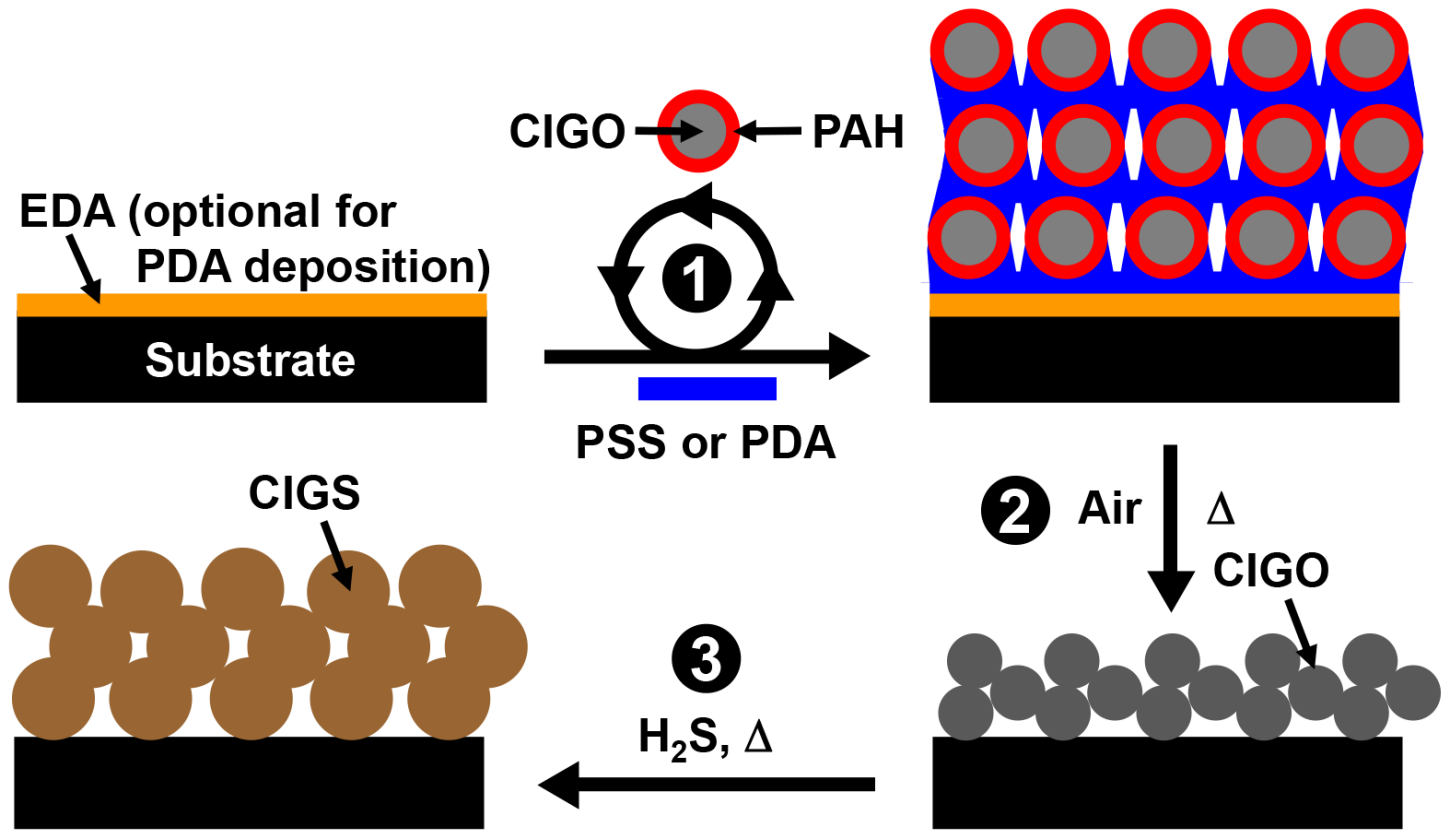
Film fabrication scheme using CIGO-PAH colloids and PSS or PDA polyelectrolytes. Process sequence (not to scale): (1) Treatment of substrate with PSS (aq) or pH 8.25 dopamine (aq) solution (for *in situ* PDA generation) followed by CIGO-PAH (aq) dispersion or CIGO-PAH/Tris pH 8.25 (aq) dispersion to deposit first bilayer of PSS/CIGO-PAH or PDA/CIGO-PAH, respectively. Repetition of treatment cycle deposits additional bilayers for multilayer film fabrication; (2) Air oxidation for 5 h at 550°C to remove organic components from CIGO particles; (3) Sulfurization for 3 h at 550°C in H_2_S to convert CIGO particles to CIGS film. Consult the Experimental Section and text for additional details.

Figure 5 illustrates the general approach for fabrication, as well as oxidation and sulfurization, of the PSS/CIGO-PAH and PDA/CIGO-PAH multilayers. As a pre-requisite for reproducible fabrication of PSS/CIGO-PAH multilayers, it was first necessary to determine appropriate substrate treatment times for reproducible deposition of CIGO-PAH and PSS layers. For this purpose, a Q-EDA/(PSS/PAH)_3_/PSS film was prepared using the 5 mg PAH⋅mL^−1^ 1.00 M NaCl (aq) and 5 mg PSS⋅mL^−1^ 1.00 M NaCl (aq) solutions as described previously. [22] Treatment of this base film with the CIGO-PAH dispersion indicated that CIGO-PAH particle deposition, as measured by 285 nm film absorbance, was >96% complete after 4 h and >98% complete after 6 h compared to a film deposited overnight (*i.e*., ≥ 12 h). Similar adsorption studies involving treatment of the CIGO-PAH layer with the 5 mg PSS⋅mL^−1^ 1.00 M NaCl (aq) solution indicated that PSS binding was complete (*i.e*., 100%) within 30 min. Consequently, substrate treatment times of 30 min and ≥ 5 h were selected for hand dipcoating depositions of the PSS and CIGO-PAH layers, respectively.

Once treatment times had been established, PSS/CIGO-PAH multilayer depositions were continued using the PSS/PAH base film to confirm layer deposition reproducibility and assess the effects of CIGO-PAH dispersion age on film growth. Film fabrication was initiated under quiescent hand dipcoating conditions using a freshly prepared stock CIGO-PAH dispersion, which aged 9 days during the time required to complete the film. The CIGO-PAH treatment dispersion was replaced by decantation with the aging CIGO-PAH stock dispersion after every 3 days of use. Care was taken not to transfer any precipitated material present into the treatment container, which was cleaned before the dispersion was replaced. Fabrication of a second film was also separately initiated after the CIGO-PAH stock dispersion was aged 2 days, with the final layers deposited using a CIGO-PAH dispersion aged 11 days.

**Figure 6:**
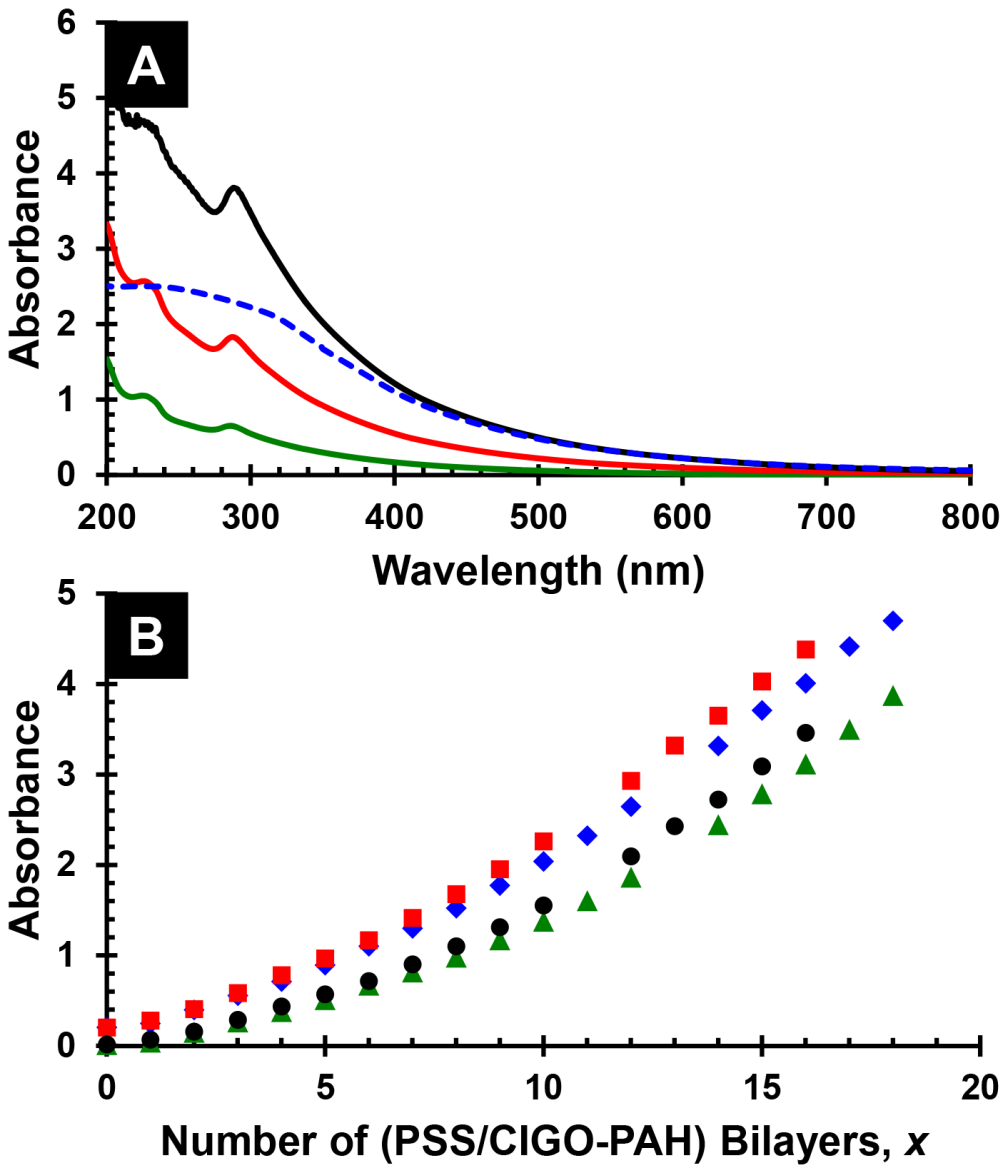
Characterization of PSS/CIGO-PAH multilayers prepared by hand dipcoating. (A) Absorbance spectra in descending order at 285 nm of PSS/CIGO-PAH multilayer films of structure Q-EDA/(PSS/PAH)_3_/(PSS/CIGO-PAH)*_x_* with *x* = 18 (black, solid), *x* = 18 after annealing in air 5 h at 550°C (blue, dashed), *x* = 12 (red, solid), and *x* = 6 (green, solid). Note that the measured absorbance represents films having the structures shown that are present on *both sides* of the quartz slide. (B) Absorbance *vs*. number of bilayers, *x*, for Q-EDA/(PSS/PAH)_3_/(PSS/CIGOPAH)*_x_* multilayers. Red squares (225 nm) and black circles (285 nm) indicate a film initiated using 2 day aged CIGO-PAH; Blue diamonds (225 nm) and green triangles (285 nm) indicate a film initiated using fresh CIGO-PAH.

Figure 6, part A, shows the absorbance spectrum of a completed 18 bilayer PSS/CIGO-PAH multilayer of structure Q-EDA/(PSS/PAH)_3_/(PSS/CIGO-PAH)_18_, together with spectra of intermediate films having 6 and 12 bilayers. Films having these structures are present on *each* side of the quartz substrate. The spectra are similar to that of the aqueous CIGO-PAH dispersion shown in Figure 2, part A, with the exception of an additional peak near 225 nm characteristic of the PSS layers. The absorbance spectrum of the 18 bilayer film after thermal annealing is also shown. The UV intensity is decreased and the PSS absorbance at 225 nm is absent after annealing due to the removal of the organic PAH and PSS components. In addition, the 285 nm peak, assigned to incompletely oxidized species within the CIGO particles, is also absent, consistent with the thermogravimetry results (*vide supra*).

Figure 6, part B, shows the changes in PSS/CIGO-PAH film absorbance, monitored at 285 nm where CIGO is the predominant absorbing species and at 225 nm where both CIGO and PSS absorb well, as a function of the number of bilayers deposited for both films. In each case, absorbance increases nonlinearly with slight upwards curvature as the number of bilayers deposited increases. The amount of material deposited is slightly but consistently larger for the film initiated using the 2-day old CIGO-PAH than the fresh CIGO-PAH dispersion. In addition, traces of settled CIGO-PAH precipitate are observed at the bottom of the CIGO-PAH containers following completion of the films, behaviors in agreement with the aggregation noted for the aqueous CIGO-PAH dispersion in the DLS studies. Our results suggest the use of CIGO-PAH dispersions aged at most 6 days, and preferably 3 days or less, to optimize linearity and reproducibility during PSS/CIGO-PAH film depositions again consistent with the DLS results.

Experiments were also performed using a robot dipcoater to demonstrate the ability to automate film fabrication directly onto our substrates for use in a manufacturing environment. Because software constraints associated with the robot limited deposition times to no more than 90 min per layer, substrate treatments were carried out with stirring to enhance deposition rates. Through some trial and error, a 90 min CIGO-PAH deposition with sample stirring (∼140±5 rpm) was found to correspond to a 6 h quiescent CIGO-PAH treatment, as measured by the 285 nm absorbance of the deposited CIGO-PAH material. However, initial attempts to directly deposit multilayers bearing more than 8 PSS/CIGO-PAH bilayers led to complete settling of the CIGO-PAH dispersion. The problem was eventually traced to cumulative carryover of small amounts PSS, identified by its characteristic UV spectrum and 225 nm absorbance band (Figure S3) into the CIGO-PAH dispersion. This apparently occurred due to incomplete draining of rinse water during rinse water refill cycles.

**Figure 7:**
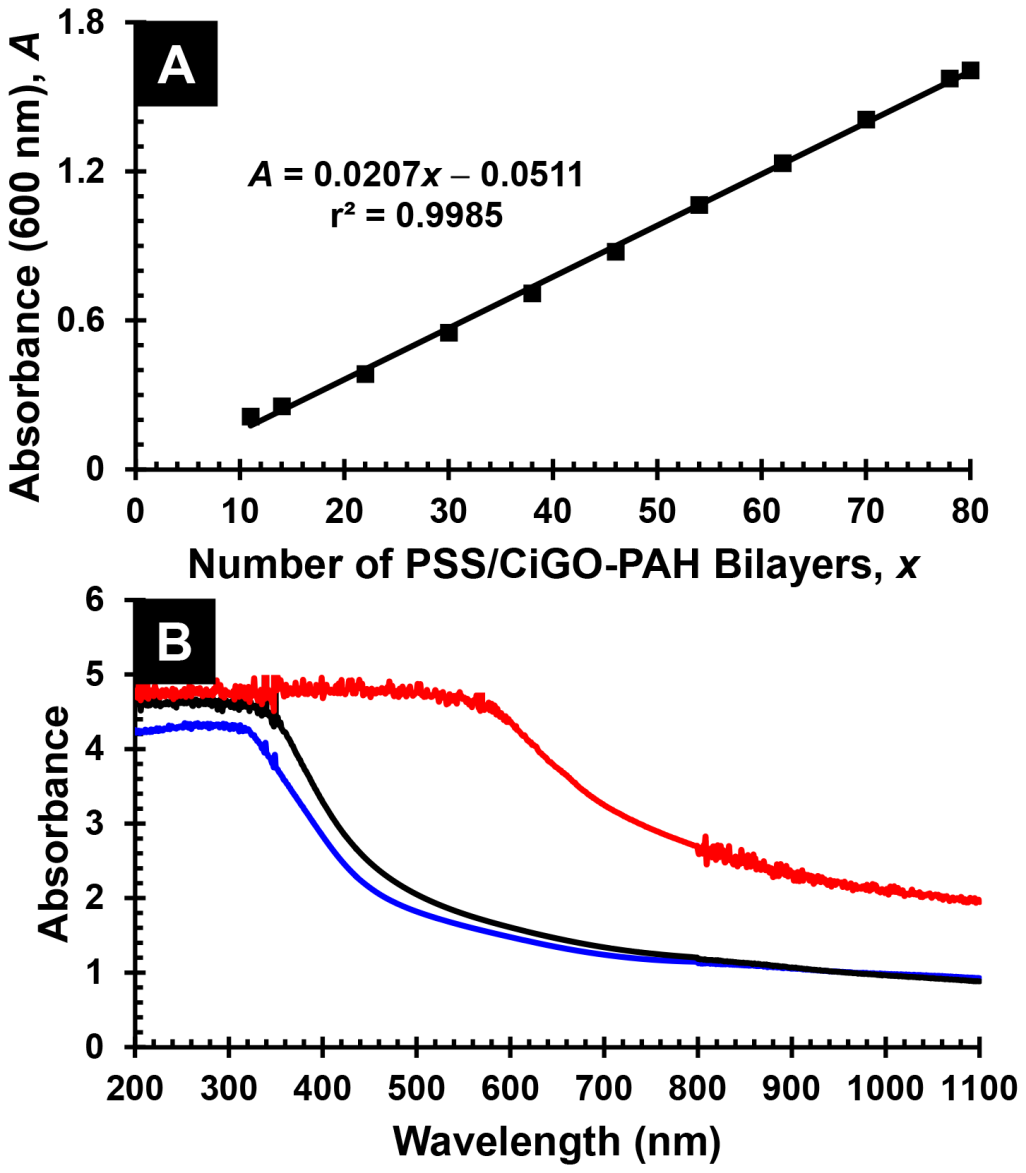
Characterization of PSS/CIGO-PAH multilayers prepared by robot dipcoating. (A) Absorbance *vs*. number of bilayers, *x*, for Q-EDA/(PSS/CIGO-PAH)*_x_* multilayers prepared via automated dipcoating using the robot. (B) Absorbance spectra in ascending order at 400 nm of robot dipcoated PSS/CIGO-PAH multilayer films of structure Q-EDA/(PSS/CIGO-PAH)_80_ after annealing in air 5 h at 550°C (blue), as deposited (black), and after H_2_S sulfurization 5 h at 550°C (red). Note that the measured absorbance derives from films having the structures shown that are present on *both sides* of the quartz slide.

Acceptable films could nevertheless be deposited if the CIGO-PAH dispersion and PSS solution were replaced after deposition of every 4−6 bilayers, provided that the sample and rinse beakers were adequately rinsed and dried before re-use. If the used CIGO-PAH dispersion was replaced with dispersion aged 3 days or less, in which aggregate levels are negligible as measured by DLS, further improvements in film quality and reproducibility accrued. Specifically, nearly linear increases in film absorbance as a function of the number of bilayers deposited were now obtained for films comprising as many as 80 bilayers (on *each* side of the quartz slide), as shown in Figure 7, part A. Subsequent oxidation and sulfurization of the film provided an absorber layer capable of complete absorption of visible and near IR light (*i.e*., absorbance >2 for all wavelengths ≤ 1100 nm), as shown in Figure 7, part B.

Attempts to prepare similar absorbers on a Mo metal substrate useful as a back electrode in photovoltaic devices revealed an additional concern. Although PSS/CIGO-PAH films were successfully deposited, coating migration onto areas of Mo not originally covered by the film was noted following sulfurization. This behavior was *not* observed for a corresponding film deposited on a quartz substrate. It suggested to us that adhesion of the PSS/CIGO-PAH multilayer to the underlying Mo substrate may be insufficient to limit film migration. In fact, an XPS study indicated that neither organothiol and organosiloxane monolayers nor polyamines were readily chemisorbed to the Mo surface (though PSS was weakly bound), behavior consistent with this hypothesis.

Therefore, we sought a polymeric replacement for PSS that could more strongly bind CIGO-PAH particles to each other and the substrate in our multilayer films. PDA is a strong adhesive [50], [51] produced by polymerization of dopamine under basic pH conditions and used by marine organisms such as mussels to anchor themselves to solid surfaces in underwater environments. [52], [53] Although its composition and structure remain uncertain, electrostatic and covalent interactions involving both the amine and catechol sites of dopamine have been identified within PDA. [54]–[56] Therefore, we expected that amine sites on our CIGO-PAH particles would be effectively bound in films containing a PDA component.

**Figure 8:**
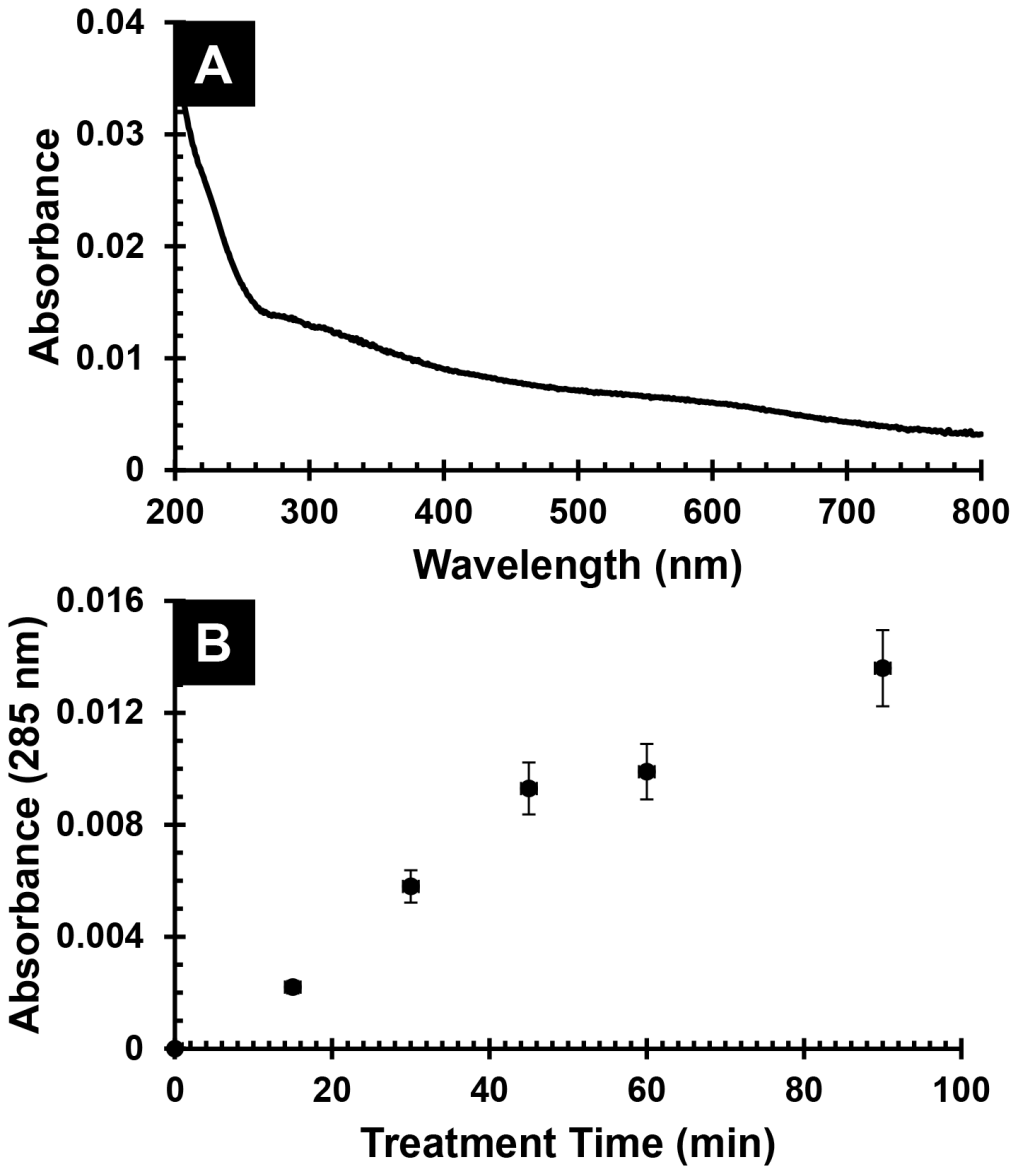
Deposition of polydopamine thin films. (A) Absorbance spectrum of polydopamine (PDA) film on a Q-EDA slide prepared by 90 min treatment in freshly made 1 mg dopamine⋅mL^−1^ 10 mM Tris pH 8.25 (aq) solution. Spectrum shown after subtraction of an untreated Q-EDA slide baseline. Note that the measured absorbance represents PDA films that are present on *both sides* of the quartz slide. (B) Time dependent absorbance at 285 nm for Q-EDA slide treated with 1 mg dopamine⋅mL^−1^ 10 mM Tris pH 8.25 (aq) buffer solution.

In fact, initial experiments revealed deposition of a yellow-brown PDA film onto Q-EDA, Si-EDA, and even Mo substrates treated for extended periods with an aqueous 1 mg dopamine⋅mL^−1^ solution containing 10 mM Tris pH 8.25 buffer, as shown in Figure 8, part A. A nearly linear deposition rate during the first 45 min was observed in Figure 8, part B, as PDA polymerization occurred on both sides of the EDA-coated quartz substrate. Thereafter, deposition continued at a slower rate (50–60%) suggesting that coverage of the EDA was complete and further reaction occurred on the already deposited PDA layer. However, attempts to bind our CIGO-PAH dispersion directly onto PDA films deposited for 45 min or longer led to irreproducible results. CIGO-PAH was reproducibly deposited only when the dispersion contained 20 mM Tris pH 8.25 buffer, indicating that the presence of catecholate anions on the PDA surface was a pre-requisite for CIGO-PAH binding. Subsequent time dependent studies indicated that CIGO-PAH particle deposition onto a PDA film was complete in as little as 45 min, a substantially shorter time than the 5 h CIGO-PAH treatments required for deposition of the corresponding PSS/CIGO-PAH films in quiescent hand dipcoating experiments.

**Figure 9:**
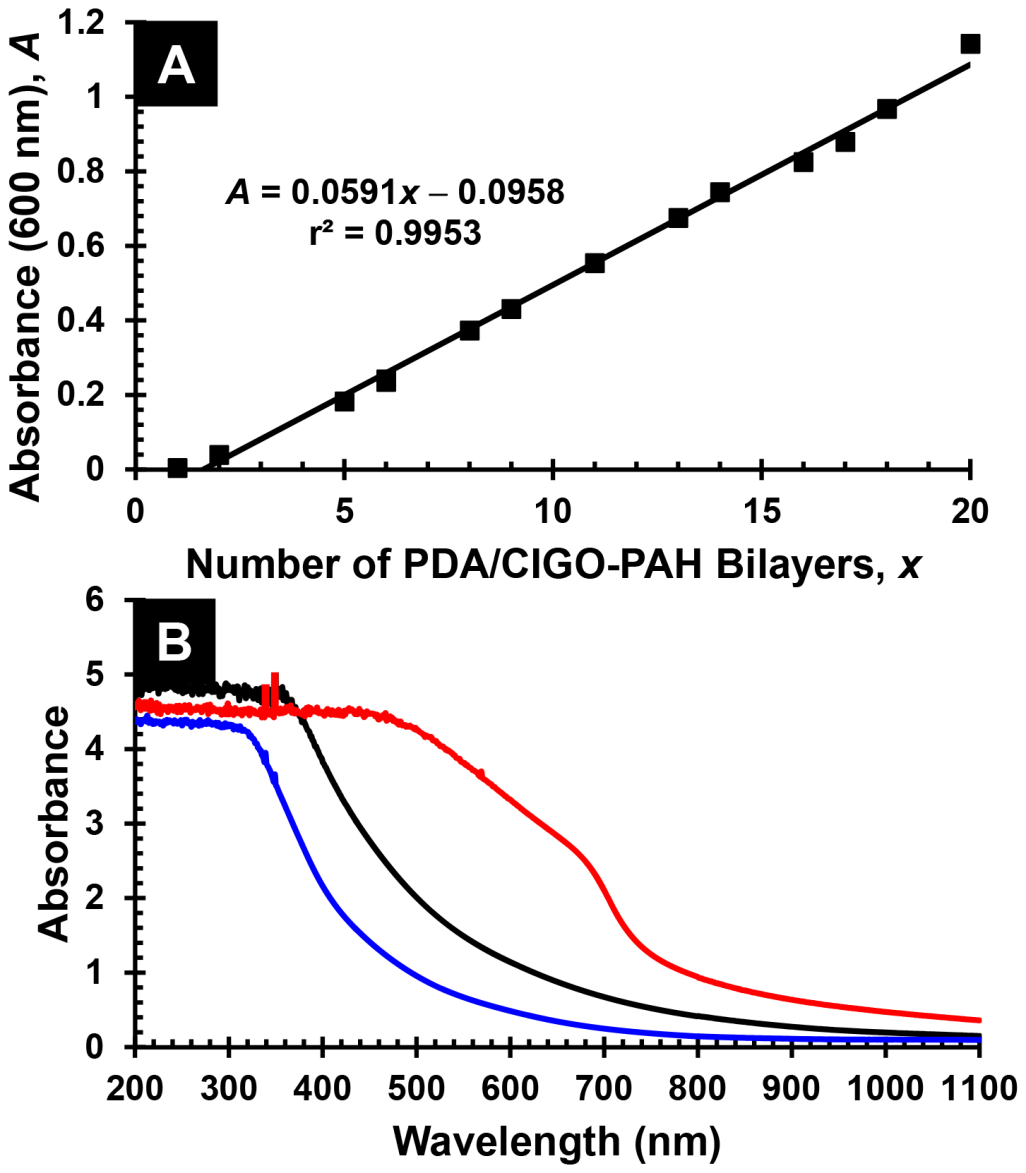
Characterization of PDA/CIGO-PAH multilayers prepared by hand dipcoating. (A) Absorbance *vs*. number of bilayers, *x*, for Q-EDA/(PDA/CIGO-PAH)*_x_* multilayers deposited by hand dipcoating. (B) Absorbance spectra in ascending order at 600 nm of hand dipcoated PDA/CIGO-PAH multilayer films of structure Q-EDA/(PDA/CIGO-PAH)_20_ after annealing in air 5 h at 550°C (blue) with thickness 953±162 nm, as deposited (black) with thickness 1163±189 nm, and after sulfurization 5 h at 550°C (red) with thickness 1079±168 nm. Film thicknesses were measured by profilometry. Note that the measured absorbance derives from films having the structures shown that are present on *both sides* of the quartz slide.

Fabrication of PDA/CIGO-PAH films directly on our substrates via quiescent hand dipcoating was successfully performed using 45 min treatments for both the PDA/Tris pH 8.25 solution and the CIGO-PAH/20 mM Tris pH 8.25 dispersion. By freshly preparing 1 mg dopamine⋅mL^−1^ 10 mM Tris pH 8.25 solution for deposition of each PDA layer and using a new aliquot of CIGO-PAH/20 mM Tris pH 8.25 dispersion, prepared by addition of 1.00 M Tris pH 8.25 buffer to 1 mg CIGO-PAH⋅mL^−1^ dispersion aged ≤ 3 days, after deposition of every 3−4 PDA/CIGO-PAH bilayers, a nearly linear and reproducible multilayer deposition was achieved per Figure 9, part A. An absorbance spectrum for the PDA/CIGO-PAH film of structure Q-EDA/(PDA/CIGO-PAH)_20_ present on *each* side of the quartz slide is shown in Figure 9, part B. Spectra are also shown in Figure 9, part B, for the corresponding oxidized and sulfurized films, which are similar to the analogous films prepared from PSS/CIGO-PAH multilayers in Figure 7, part B.

**Figure 10:**
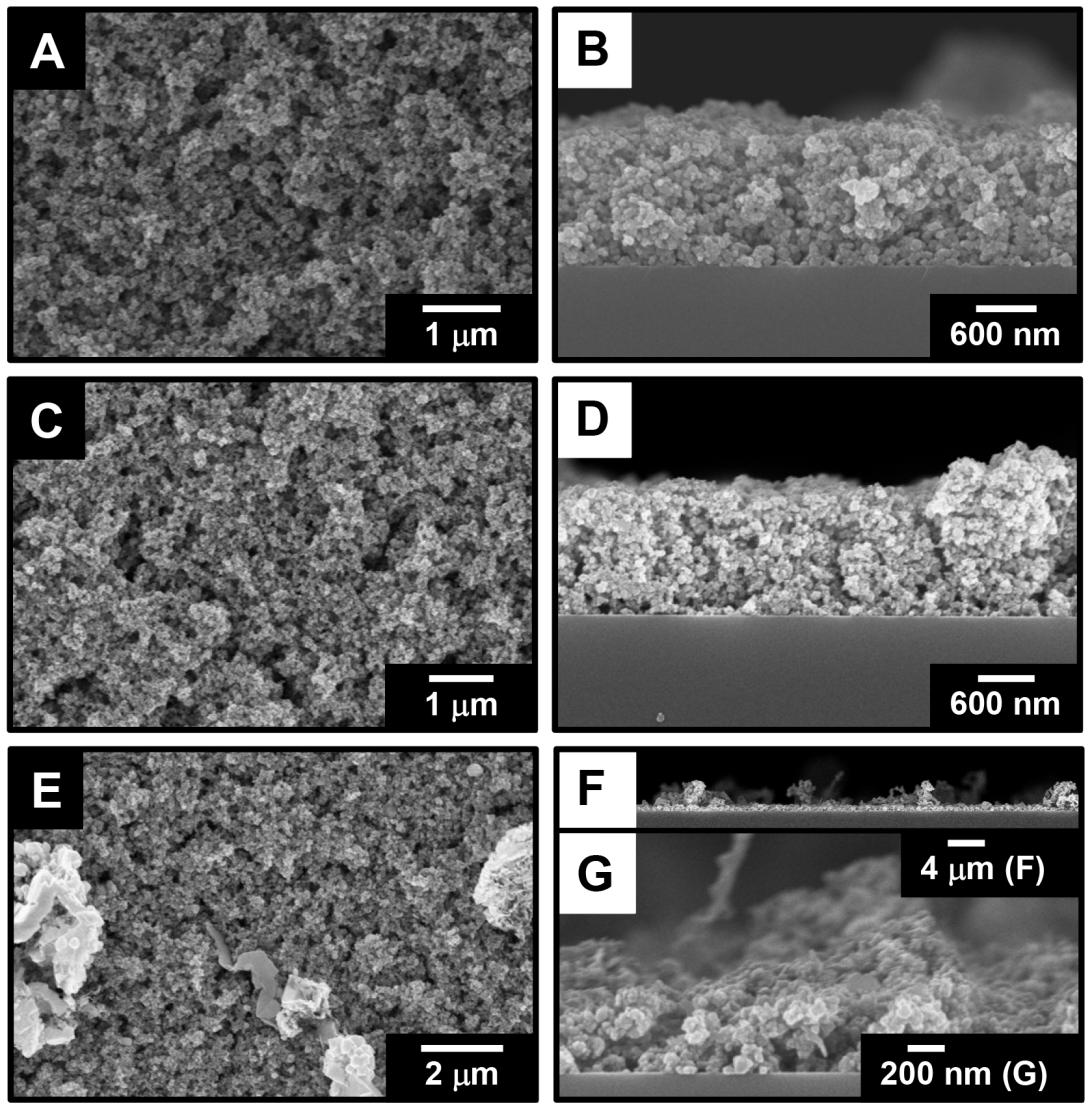
SEM images of Si-EDA/(PDA/CIGO-PAH)_26_/PDA films. Top-views (A, C, E) and side-views (B, D, F, G) for as-deposited film (A, B), as-deposited film after 5 h air oxidation at 550°C (C, D), and oxidized film after sulfurization 3 h in H_2_S at 550°C (E, F, G) are shown.

Somewhat thicker films of structure Si-EDA/(PDA/CIGO-PAH)_26_/PDA were also deposited via hand dipcoating on Si-EDA wafers for characterization of film morphology and topography. Top-view and side-view SEM images of the Si-EDA/(PDA/CIGO-PAH)_26_/PDA film as deposited and after oxidation and sulfurization are shown in Figure 10. The as-deposited film in Figure 10, part A, reveals a sponge-like morphology comprising aggregates of ca. 125 nm diameter CIGO-PAH particles cemented together by PDA. The side-view of the film shown in Figure 10, part B, indicates that nanochannels having diameters comparable to the CIGO-PAH particle sizes are present and completely penetrate the film, consistent with inefficient packing of the particles during the deposition process. Film thickness is ca. 1500 nm, with a roughness of ±150 nm again consistent with the presence of pore structures and inefficient particle packing. Although film thickness and roughness (*i.e*., ca. 1300±100 nm) are each reduced somewhat following air oxidation to remove the PDA and PAH components (Figure 10, parts C and D), porosity, particle size, and general film morphology are little changed.

However, significant changes are observed following sulfurization. Lighter regions of coalesced material are clearly observed, together with ca. 100 nm particles and pores, on the top surface of the film following sulfurization in Figure 10, part E. The side-view of the same film in Figure 10, part F, shows that the lighter material comprises coiled and rod-shaped fibrils several microns in height distributed over the top surface of the film. A close-up of the film side-view in Figure 10, part G, indicates that the film has thinned significantly, with film thickness in regions absent the fibrils now just ca. 600±100 nm and nanochannels penetrating the film to the underlying Si substrate clearly seen. These observations are collectively consistent with a redistribution of material during the sulfurization process, as also noted during sulfurization of the PSS/CIGO-PAH films deposited on Mo substrates (*vide supra*). The phenomenon is clearly associated with the presence of the substrate and/or polyelectrolytes, since as-prepared CIGO particles are cleanly sulfurized to CIGS (Figure 1, part B) without the dramatic morphology changes observed here. However, the mechanism associated with the film transformation during sulfurization is currently unknown and remains under investigation.

**Figure 11:**
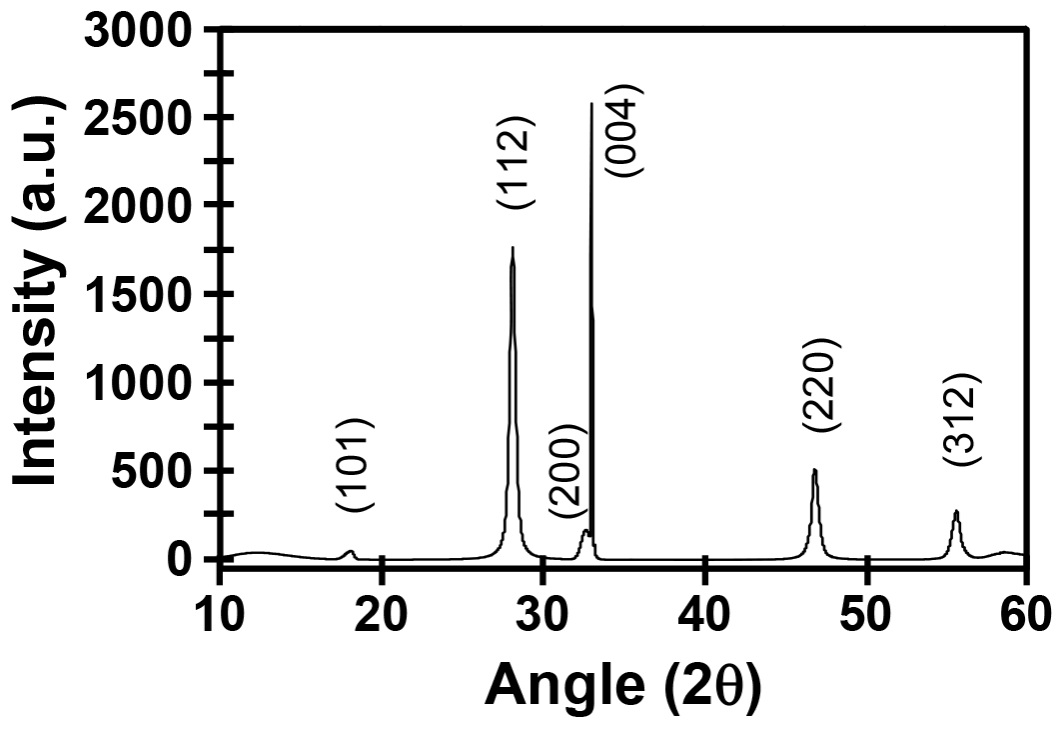
XRD of the CIGS film shown in Figures 9E–G. Consult the text for additional details and discussion.

The composition of the sulfurized film shown in Figures 10, parts E–G, is verified by the XRD analysis in Figure 11, which exhibits the reflection peaks expected for CIGS material. No evidence for additional phases or materials is observed, indicating that the fibrils and top surface particulates comprise the same material. This is further confirmed by EDS analyses performed on both the particulate and fibrillar regions (Figure S4). The former exhibits an elemental composition (in atom %) of Cu = 18.35%, In = 21.05%, Ga = 5.07%, and S = 45.26%. A 10.27% oxygen value is also observed, consistent with sampling of the underlying substrate oxide through the film nanochannels during the measurement. EDS of the fibrillar regions provides a substantially identical composition of Cu = 18.73%, In = 20.87%, Ga = 5.50%, and S = 47.10% (with O = 7.81%). Compared to the target CIGS composition of Cu = 25%, In = 17.5%, Ga = 7.5%, and S = 50% expected from our CIGO precursor of composition CuIn_0.7_Ga_0.3_O_2_, our CIGS material is indium rich but both Cu and Ga poor. This result is consistent with our previous observation of a blue-green PAH supernatant characteristic of mixed chloro- and amine- complexes of Cu(II) [57] following CIGO particle sonication in PAH (Figure S5), suggesting that the as-prepared CIGO particle composition may need to be adjusted during FSP to account for selective metal ion extraction during polyelectrolyte binding.

Finally, we note that attempts to utilize CIGS films similar to those shown in Figure 10, parts E–G, as light absorber components in photovoltaic devices fail to produce a working device, despite the use of thicker precursor PDA/CIGO-PAH films to ameliorate the effects of film thinning and the presence of nanochannels noted in Figure 10, part G. Specifically, a photovoltaic device fabricated from a 46 bilayer PDA/CIGO-PAH film on Mo as described in the Experimental Section (Figure S6) exhibits a short circuit response. Such behavior indicates that the drastic morphology changes that accompany sulfurization of our oxidized polyelectrolyte/CIGO-PAH multilayer films, as described and prepared here, remain an impediment to their successful use as light absorber layers in photovoltaic devices.

## Conclusions

We have described procedures for the FSP preparation of gram scale quantities of CIGO particles having diameters of 10−75 nm and tunable compositions from simple solution precursors. Subsequent binding of polyallylamine (PAH) to the particle surface via sonication is accompanied by limited extraction of Cu and Ga from the particle surface and particle aggregation, with the resulting ca. 125 nm diameter CIGO-PAH species forming aqueous colloidal dispersions whose stabilities depend on dispersion age and pH. Dispersions are sufficiently stable, however, for reproducible fabrication of composite PSS/CIGO-PAH multilayer films with PSS via a layer-by-layer hand dipcoating approach. Automation of the process using a robot dipcoater has been demonstrated, providing films containing as many as 80 PSS/CIGO-PAH absorbing >90% of all light of λ ≤ 1100 nm after oxidation and sulfurization. The analogous PDA/CIGO-PAH multilayers are also readily prepared from the CIGO-PAH dispersions via hand-dipcoating using PDA, generated *in situ* by dopamine polymerization, rather than PSS.

Both the PSS/CIGO-PAH and PDA/CIGO-PAH multilayer films are readily oxidized in air to corresponding CIGO films and sulfurized to form CIGS films. The oxidized films retain morphology similar to that of the original multilayers, characterized by fused ca. 125 nm diameter particles and tortuous nanochannels indicative of inefficient particle packing during film deposition. However, drastic morphological changes during film sulfurization lead to the appearance of micron-scale surface fibrils and corresponding thinning of the CIGS film, exposing the underlying substrate via the nanoscale channels present in the film and rendering such films unsuitable for use as light absorber layers in photovoltaic devices.

Optimization of our process to provide more compact CIGS films useful as photovoltaic absorber layers will require addressing both the particle packing issues responsible for film channels and material redistribution phenomenon during sulfurization that promote photovoltaic device short circuit behavior. With regard to packing issues, the use of smaller colloidal particles, produced via jet milling or partial chemical dissolution (*e.g*., HCl in Experimental Section, *vide supra*) of existing as-deposited CIGO particles (or CIGS particles prepared from them, Figure 1, part B), is expected to provide more uniformly packed films devoid of channels. Film quality may also benefit from enhanced nanoparticle packing via gentle ultrasonic agitation during film deposition [58] and internal restructuring and smoothing of films containing polyelectrolyte components via post-deposition salt annealing. [59].

In the absence of a more detailed understanding of the drastic film morphology changes accompanying our sulfurization process, preparation of LbL films using CIGS particles prepared from the as-deposited CIGO particles are clearly preferred as an alternative fabrication scheme to avoid our CIGO film sulfurization processing step. In fact, preliminary experiments in our laboratory indicate that the binding of polyamines demonstrated for CIGO particles is also applicable for CIGSe particles, as required for fabrication of multilayer films using the LbL approach described here. Additionally, during the course of our work two reports have appeared that further support the viability of this approach. Specifically, Shrestha and coworkers [34] have described a LbL process in which 10 nm diameter CIGSe particles stabilized by bound oleylamine ligands were successfully coated by PSS and, together with PEI, used to fabricate a closely packed PEI/CIGSe-PSS multilayer film. Use of their film as an absorber layer in a photovoltaic device *without* prior thermal annealing to remove the organic components provides a cell exhibiting a solar conversion efficiency of 3.5%. In addition, Korgel and coworkers [17] have shown that thermal annealing under *inert* atmosphere to remove oleylamine adsorbate from CIGSe (*i.e*., Cu_0.8_In_0.7_Ga_0.3_Se_2_) nanocrystals in spraycoated nanocrystal films does not materially affect nanocrystal composition and promotes subsequent nanocrystal sintering required for optimization of photovoltaic device performance. We are currently exploring and adapting such techniques in our multilayer systems to fabricate improved quality CIGS films via the LbL approach using our more abundant CIGO particles, or CIGS particles prepared from them, as low cost scalable materials.

## Supporting Information

Figure S1 **Thermogravimetric temperature ramp scans.** (A) As-prepared CIGO particles. (B) CIGO-PAH particles.
(TIF)

Figure S2 **Stability of the CIGO-PAH dispersion.** Additional DLS data are shown concerning the stability of the stock 1 mg CIGO-PAH⋅mL^−1^ (aq) dispersion (without added Tris pH 8.25 buffer). Only the major peaks are detected. No particles >300 nm are seen at the time points shown: (A) aged 1 day; (B) aged 2 days; (C) aged 6 days (not mixed prior to measurement).
(TIF)

Figure S3 **PSS contamination of the CIGO-PAH dispersion during robot-dipcoated PSS/CIGO-PAH multilayer deposition.** The absorbance spectrum of the supernatant remaining after settling of the CIGO-PAH treatment dispersion following deposition of 8 PSS/CIGO-PAH bilayers on a Q-EDA slide using the robot dipcoater is shown. Residual PSS is detected in the solution by means of its characteristic absorbance at 225 nm and adsorption to traces of remaining suspended CIGO-PAH particles. Cuvette pathlength = b = 0.10 cm *vs*. water blank baseline.
(TIF)

Figure S4 **EDS spectra of CIGS film of** **Figure 9E** **in the main article text.** (A) Porous particulate (darker) regions. (B) Coalesced fibrillar (lighter) regions.
(TIF)

Figure S5 **Evidence for reaction of PAH with CIGO components during sonication.** The absorbance spectrum of the blue-green 5 mg PAH⋅mL^−1^ 1.00 M NaCl (aq) supernatant remaining after sonication with as-prepared CIGO particles and removal of the CIGO-PAH reaction product via centrifugation is shown. Cuvette pathlength = b = 0.10 cm *vs*. water blank baseline. Consult the main article text for additional details and discussion.
(TIF)

Figure S6 **Schematic of the photovoltaic test device (not to scale).** The thin MoS_2_ film present at the Mo-Cu(In,Ga)S_2_ interface is omitted for clarity. Consult the Experimental Section for further details.
(TIF)

Table S1 **Effect of Tris pH 8.25 buffer on the CIGO-PAH dispersion stability.**
(DOC)
