# Supplementary figures and images for: Preparation and Layer-by-Layer Solution Deposition of Cu(In,Ga)O2 Nanoparticles with Conversion to Cu(In,Ga)S2 Films
Source: PLoS One. 2014 Jun 18;9(6):e100203. doi: 10.1371/journal.pone.0100203 (PMC4062496; doi:10.1371/journal.pone.0100203)

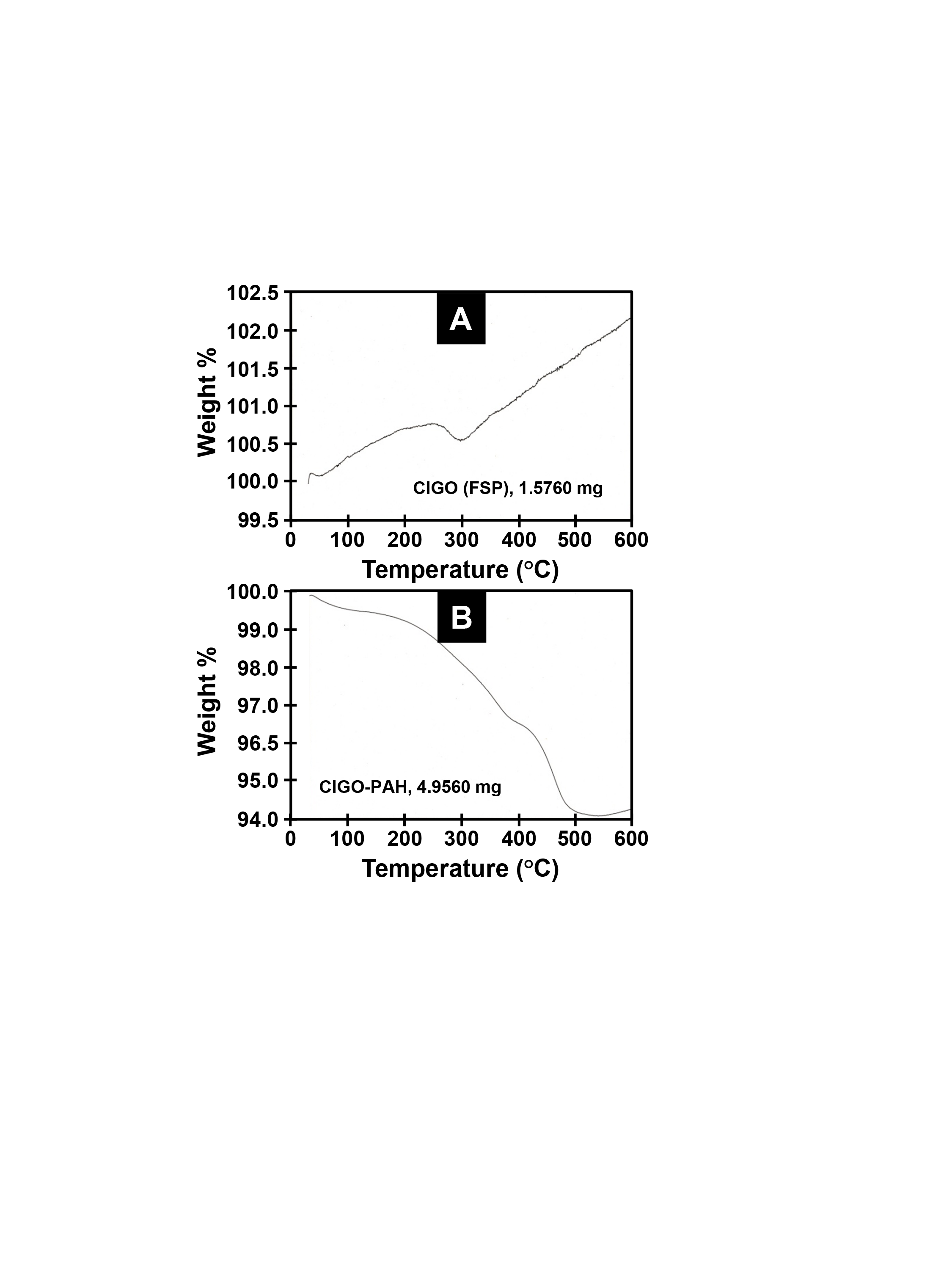

Supplement: Figure S1 — Thermogravimetric temperature ramp scans. (A) As-prepared CIGO particles. (B) CIGO-PAH particles. (TIF) [file pone.0100203.s001.tif]

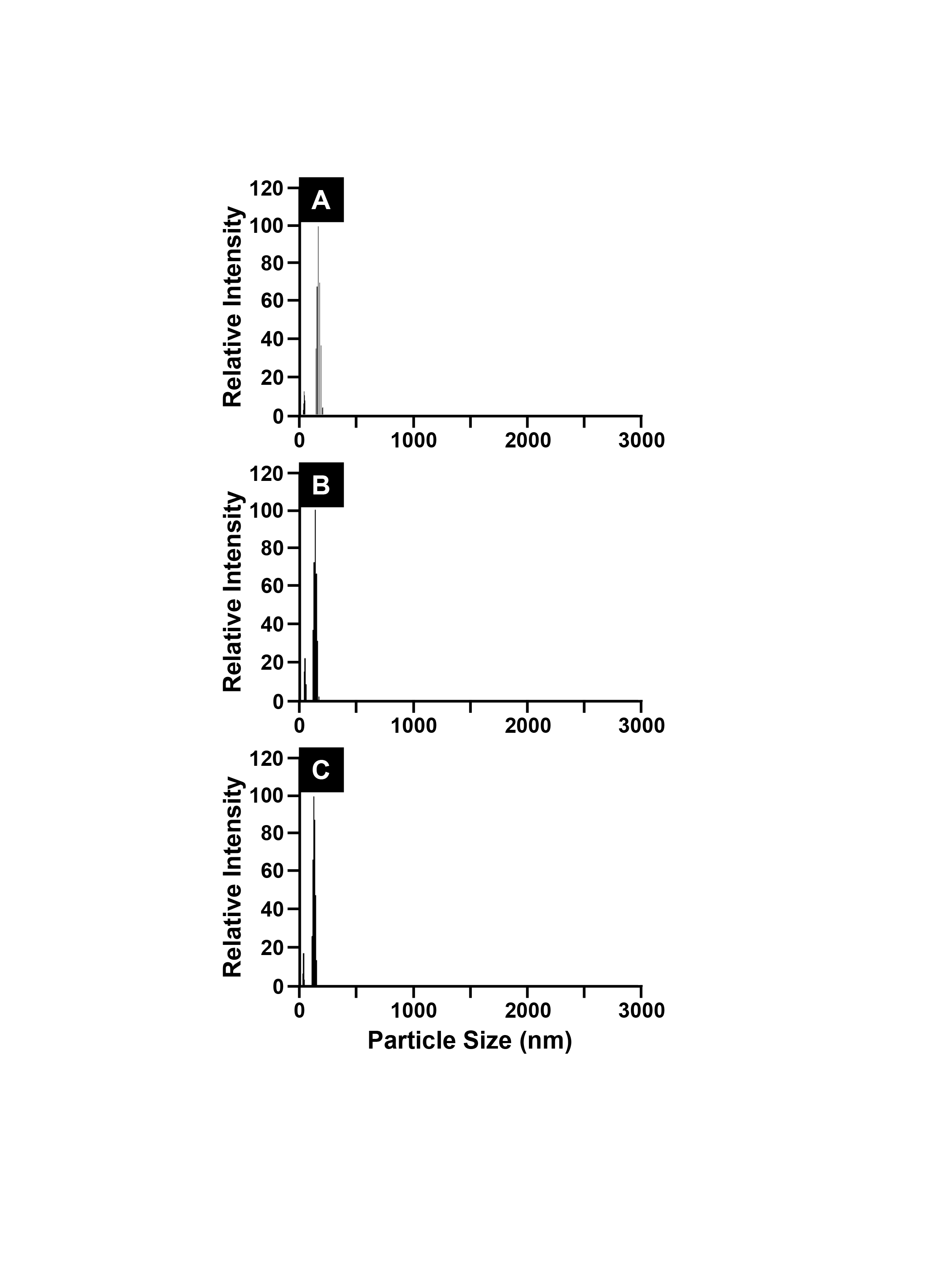

Supplement: Figure S2 — Stability of the CIGO-PAH dispersion. Additional DLS data are shown concerning the stability of the stock 1 mg CIGO-PAH⋅mL−1 (aq) dispersion (without added Tris pH 8.25 buffer). Only the major peaks are detected. No particles >300 nm are seen at the time points shown: (A) aged 1 day; (B) aged 2 days; (C) aged 6 days (not mixed prior to measurement). (TIF) [file pone.0100203.s002.tif]

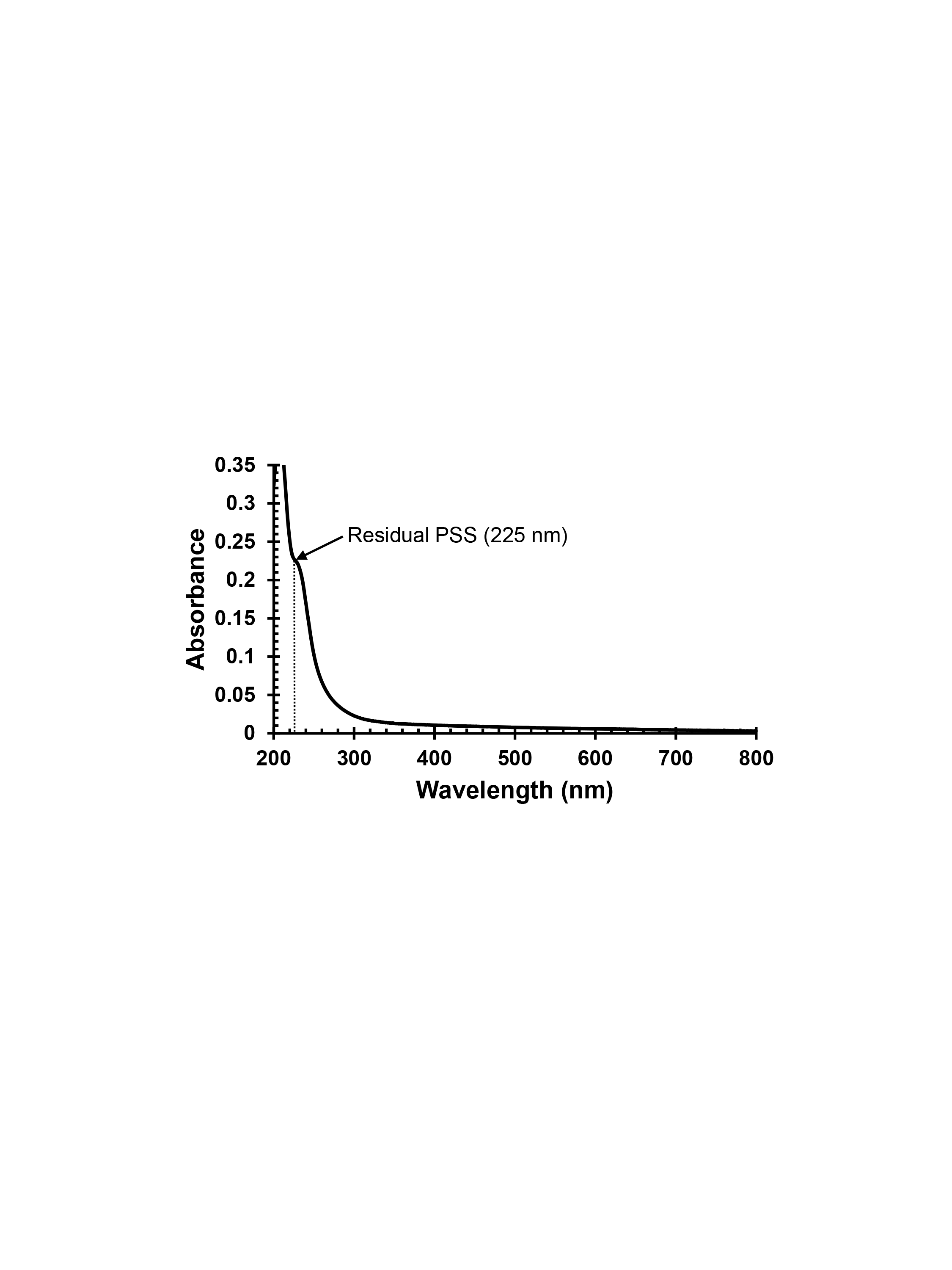

Supplement: Figure S3 — PSS contamination of the CIGO-PAH dispersion during robot-dipcoated PSS/CIGO-PAH multilayer deposition. The absorbance spectrum of the supernatant remaining after settling of the CIGO-PAH treatment dispersion following deposition of 8 PSS/CIGO-PAH bilayers on a Q-EDA slide using the robot dipcoater is shown. Residual PSS is detected in the solution by means of its characteristic absorbance at 225 nm and adsorption to traces of remaining suspended CIGO-PAH particles. Cuvette pathlength = b = 0.10 cm vs. water blank baseline. (TIF) [file pone.0100203.s003.tif]

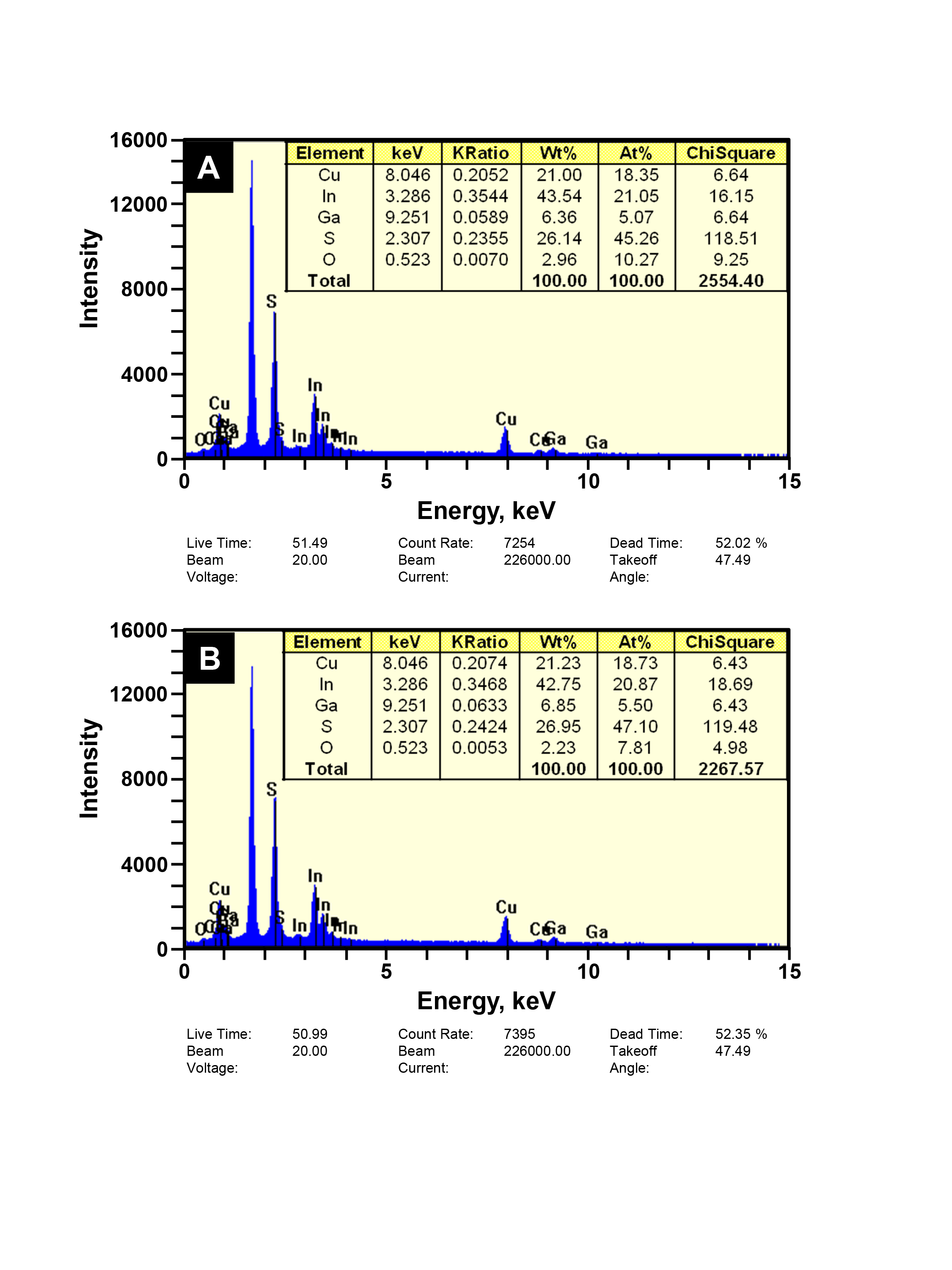

Supplement: Figure S4 — EDS spectra of CIGS film of Figure 9E in the main article text. (A) Porous particulate (darker) regions. (B) Coalesced fibrillar (lighter) regions. (TIF) [file pone.0100203.s004.tif]

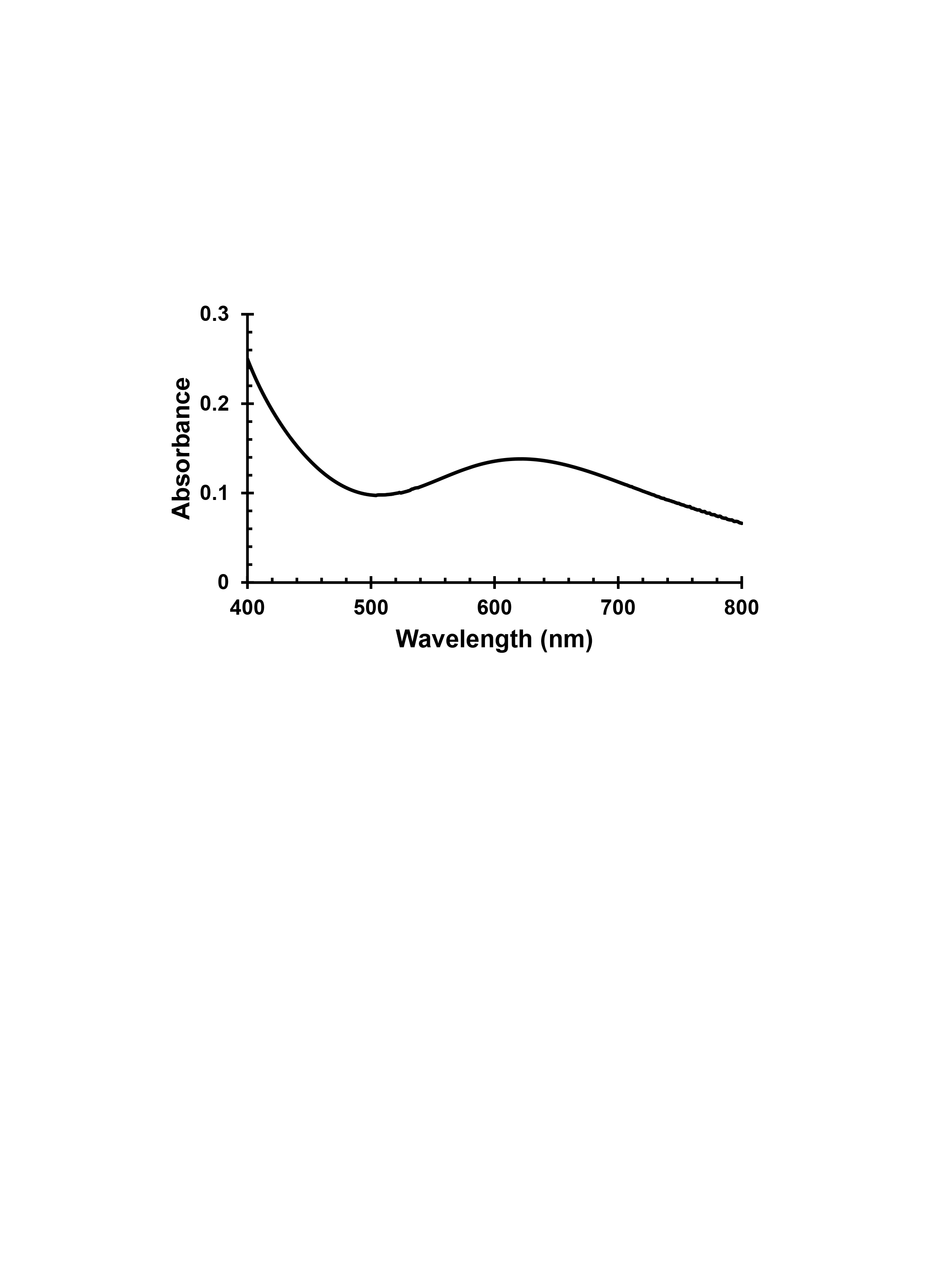

Supplement: Figure S5 — Evidence for reaction of PAH with CIGO components during sonication. The absorbance spectrum of the blue-green 5 mg PAH⋅mL−1 1.00 M NaCl (aq) supernatant remaining after sonication with as-prepared CIGO particles and removal of the CIGO-PAH reaction product via centrifugation is shown. Cuvette pathlength = b = 0.10 cm vs. water blank baseline. Consult the main article text for additional details and discussion. (TIF) [file pone.0100203.s005.tif]

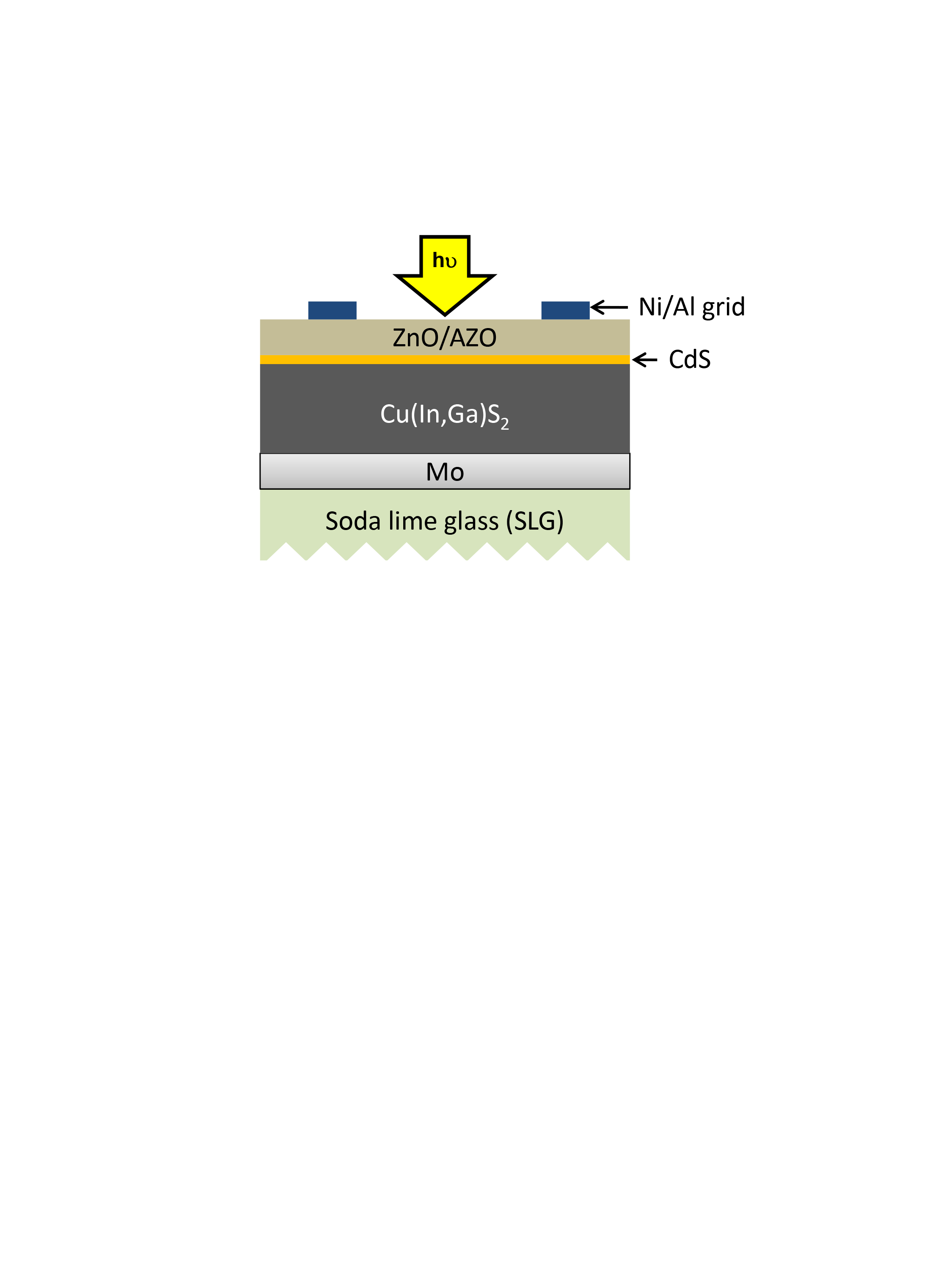

Supplement: Figure S6 — Schematic of the photovoltaic test device (not to scale). The thin MoS2 film present at the Mo-Cu(In,Ga)S2 interface is omitted for clarity. Consult the Experimental Section for further details. (TIF) [file pone.0100203.s006.tif]
