# Supplementary material for: Preparation and Layer-by-Layer Solution Deposition of Cu(In,Ga)O2 Nanoparticles with Conversion to Cu(In,Ga)S2 Films
Source: PLoS One. 2014 Jun 18;9(6):e100203. doi: 10.1371/journal.pone.0100203 (PMC4062496; doi:10.1371/journal.pone.0100203)
Supplement: Table S1 — Effect of Tris pH 8.25 buffer on the CIGO-PAH dispersion stability. (DOC) [file pone.0100203.s007.doc]

**Table S1:** Effect of Tris pH 8.25 buffer on the CIGO-PAH dispersion stability.

| **Time, min**^1^ | **Particle Size, nm**^2^ **(Relative Intensity)^3^** |
| --- | --- |
| 30 | 242 (13), 274 (13), 311 (20), 353 (13), 400 (10), 454 (2) |
| 50 | 217 (32), 486 (3), 875 (3), 1018 (3), 1184 (3), 1994 (3) |
| 60 | 211 (13) |
| 110 | 492 (6), 572 (6), 665 (6), 774 (5), 899 (3), 1045 (3) |
| 130 | 265 (7), 300 (17), 341 (33), 387 (23), 439 (12) |
| 200 | 222 (58), 250 (57), 281 (27), 316 (10) |
| 220 | 671 (7), 1966 (2) |
| 250 | 217 (24), 241 (30), 268 (38), 297 (21), 330 (10) |
| 320 | 221 (44), 267 (15) |
| 420 | 1439 (3), 1675 (4), 1950 (3), 2271 (2) |

**^1^** Time elapsed after addition of Tris pH 8.25 (aq) buffer solution to a freshly prepared 1 mg CIGO-PAH⋅mL^−1^ (aq) dispersion. The final concentration of Tris pH 8.25 buffer in the dispersion is 20 mM.

**^2^** Particle size in nm as determined from DLS measurements. Only particles > 200 nm are shown.

**^3^** Intensity of the DLS peak corresponding to each particle size normalized relative to the major peak set as relative intensity = 100.
